# Long-read sequencing and de novo genome assembly of marine medaka (*Oryzias melastigma*)

**DOI:** 10.1186/s12864-020-07042-7

**Published:** 2020-09-16

**Authors:** Pingping Liang, Hafiz Sohaib Ahmed Saqib, Xiaomin Ni, Yingjia Shen

**Affiliations:** 1 College of the Environment and Ecology, Xiamen University, Xiamen, 361102 China; 2 State Key Laboratory of Ecological Pest Control for Fujian and Taiwan Crops, Fujian Agriculture and Forestry University, Fuzhou, 350002 China; 3 Fudan University, Shanghai, 200240 China

**Keywords:** de novo genome assembly, Marine ecotoxicology, Pacific biosciences SMRT sequencing, Transposable elements

## Abstract

**Background:**

Marine medaka (*Oryzias melastigma*) is considered as an important ecotoxicological indicator to study the biochemical, physiological and molecular responses of marine organisms towards increasing amount of pollutants in marine and estuarine waters.

**Results:**

In this study, we reported a high-quality and accurate de novo genome assembly of marine medaka through the integration of single-molecule sequencing, Illumina paired-end sequencing, and 10X Genomics linked-reads. The 844.17 Mb assembly is estimated to cover more than 98% of the genome and is more continuous with fewer gaps and errors than the previous genome assembly. Comparison of *O. melastigma* with closely related species showed significant expansion of gene families associated with DNA repair and ATP-binding cassette (ABC) transporter pathways. We identified 274 genes that appear to be under significant positive selection and are involved in DNA repair, cellular transportation processes, conservation and stability of the genome. The positive selection of genes and the considerable expansion in gene numbers, especially related to stimulus responses provide strong supports for adaptations of *O. melastigma* under varying environmental stresses.

**Conclusions:**

The highly contiguous marine medaka genome and comparative genomic analyses will increase our understanding of the underlying mechanisms related to its extraordinary adaptation capability, leading towards acceleration in the ongoing and future investigations in marine ecotoxicology.

## Background

With the rapid development of global industrialization, pollutants, such as oil contaminations and heavy metals, released into the rivers and coastal waters increase every year [1–3]. Those pollutions have drawn extensive attention because they are toxic, non-biodegradable, easy to accumulate and they have drastic effects on living organisms and the ecosystem. Furthermore, the ecotoxicological impacts of pollutants are different on inhabiting flora and fauna between seawater and freshwater ecosystems [4, 5]. Whereas, many characteristics of seawater are dramatically different from those of freshwater (i.e., ionic strength, buoyancy, salinity, density, dissolved oxygen and pH) [6, 7]. These differences modulate the impact of ecotoxicological features of pollutants, such as the packing fraction and size, the bioaccumulation of the pollutants, the distribution and composition of the pollutants in liquid and solid phases [8]. Thus, the rising level of anthropogenic pollutants in coastal and estuaries waters is attracting researchers to establish an appropriate seawater model organism to precisely examine the ecotoxicological effects of contaminants on evolutionary adaptations of marine fauna.

Over the past decades, several fish species such as tilapia (*Oreochromis niloticus*) [9], rainbow trout (*Oncorhynchus mykiss*) [10], Japanese medaka (*Oryzias latipes*) [11] and zebrafish (*Danio rerio*) [12] have been widely used as model organisms to study the ecotoxicological impacts on freshwater ecosystems in laboratory experiments. Researchers have found that some coastal or estuaries candidate species; such as *Enteromorpha linza* [13], *Corophium acherusicum* [14] and *Ctenogobius giurinus* [15], can potentially be used for the ecotoxicological investigations in seawater ecosystems. However, research findings based on these seawater species lag far behind than their freshwater counterparts because of high species specificity to the living environment and the lack of adequate genetic information [16]. Consequently, researchers are in urgent need of marine sentinel model organisms, as many estuaries and coastal waters are highly contaminated.

The marine medaka, *Oryzias melastigma,* also designated as *O. dancena*, distributes broadly in the coastal and fresh waters of Pakistan, India, Myanmar and Thailand [17]. *O. melastigma* is considered as a pragmatic model fish due to its smaller size (4.5 to 23 mm), short life span (2–3 months), high fecundity, distinctive life stages, prominent gender dimorphism in the morphology of anal fin [18] and adaptability to survive in varying aquatic salinity, ranging 0–35 ppt [4, 5]. These physical and morphological characteristics have made the *O. melastigma* a model organism for ecotoxicological investigations [16, 19–24]. In recent years, many ecotoxicological studies have been focused on molecular responses of *O. melastigma* against several environmental stresses [4]. However, previous methodologies or sequencing technology had limitations that need to be amended for correct demonstration of genomes and the better understanding of molecular adaptations.

Fortunately, plummeting cost and numerous advancements in sequencing technologies and bioinformatic algorithms have made assembling of highly sophisticated genomes possible with relatively low cost. Currently, one draft genome of *O. melastigma* has published based on a reference genome assistant assembling approach [25]. The published genome of *O. melastigma* was generated using Illumina reads from several libraries, including three paired-end libraries (PE400, PE500 and PE800) and four MP (mate-pair) libraries (MP2kb, MP5kb, MP10kb and MP20kb). Then scaffolds and pseudo-chromosomes were assembled based on alignment to the chromosomes of Japanese medaka (*Oryzias latipes*) genome [25]. However, studies have shown that the usage of short Illumina sequencing reads for whole-genome sequencing is a cost-efficient way, but it can also omit the most exciting and perhaps evolutionarily important genome regions [26]. Moreover, duplicated regions of the genome are too tricky to assemble due to their high sequences identity and repetitive nature [27–29]. Therefore, the recently duplicated and high repetitive regions in the previous genome assembly of *O. melastigma* may collapse characteristically. Because using only short Illumina sequencing reads is futile to assemble the duplicated and repetitive “dark-matter” regions of the genome.

Long-read genome sequencing is a more promising approach that provides high consensus accuracy, long reads length, low level of bias and simultaneous epigenetic characterization of complex vertebrate genomes [29–33]. These advantages of long-read based genome sequencing make it a useful tool for the whole genome sequencing, analyses of hard-to-sequence regions in complex genomes, targeted sequencing, evolutionary and phylogenetic relationship analyses of complex populations and epigenetic characterizations [34, 35]. Therefore, this study was carried out; i) to emphasize the use of long-read sequencing in molecular-based ecotoxicological studies by correct assembling of recently duplicated regions, filling gaps and characterize the high repetitive regions in the genome of *O. melastigma,* Furthermore, ii) to apply the comparative genomics and phylogenetic relationship approaches to understand the origin and evolutionary adaptations of *O. melastigma*. This study will provide a high-quality genome assembly and better understandings of the underlying evolutionary mechanisms by which *O. melastigma* has adapted to diverse living environments.

## Results

### Genome sequencing, assembly and annotation

The genome of adult male marine medaka was sequenced using a combination of several sequencing approaches. The primary genome assembly of *O. melastigma* was generated using single-molecule real-time (SMRT) sequencing (PacBio Sequel), Illumina paired-end sequencing (Illumina HiSeqX ten) and 10X Genomics linked-reads. The whole-genome size of *O. melastigma* was estimated to be ~ 855 Mb by k-mer analysis (Additional file 2: Table S1). We assembled the *O. melastigma* genome using 80.26X long-read coverage of PacBio sequencing data (68.61Gb) (Additional file 2: Table S2). The sequenced reads were self-corrected and the resulting genome assembly consisted of 2610 contigs (with contig N50 of 700 kb), yielding a high-quality consensus sequence with a total length of ~ 835 Mb. Then, the contigs were connected to scaffolds by 10X Genomics linked-read data. Finally, Illumina paired-end sequencing data was used for error correction (Additional file 2: Table S2). The total size of the assembly was 835.41 Mb in the contig level (with contig N50 of 707.80Kb), and the total length of the final assembly was 844.17 Mb with the most extended scaffold reaching up to 8.67 Mb (39.31% GC-contents and obtained 1257 scaffolds with a scaffold N50 of 1.71 Mb) (Table 1).

**Table 1:** Assembly statistics of new *O. melastigma* assembly

Sample ID	Length		Number	
	Contig (bp)	Scaffold (bp)	Contig	Scaffold
Total	835,406,597	844,166,318	2588	1257
Max	5,175,882	8,672,543	–	–
Number > =2000	–	–	2572	1241
N50	707,795	1,709,016	314	151
N60	537,651	1,265,745	449	208
N70	399,374	1,001,150	629	283
N80	271,770	678,245	881	385
N90	157,232	413,071	1281	543

To evaluate the accuracy of the genome, we mapped paired-end sequence data generated by Illumina HiSeqX ten platform to the *O. melastigma* genome with BWA (Burrows-Wheeler Aligner) [36]. The 96.19% mapping rate and 99.15% coverage rate showed a high consistency between reads and the genome assembly (Additional file 2: Table S3). Furthermore, we performed a variant calling using SAMtools [37] to evaluate the accuracy of the genome at the single-base level. We identified a total of 3,785,501 single-nucleotide polymorphisms (SNPs) (0.47% of the genome). The 28,611 SNPs (0.0036% of the genome) belonged to homozygous single-nucleotide polymorphisms (Additional file 2: Table S4), indicating high accuracy of genome assembly at the single-base level. Half of the total SNPs were located in the genic regions, with about 5% were distributed in the exon regions (Additional file 2: Table S5).

To assess the completeness of the marine medaka assembly, we compared the assembly to the established core vertebrate gene sets by two methods, Benchmarking Universal Single-Copy Orthologs (BUSCO) [38] and Core Eukaryotic Genes Mapping Approach (CEGMA) [39]. BUSCO and CEGMA analysis identified 94.90% of the eukaryotic BUSCO conserved gene set, and 96.37% of CEGMA gene sets entirely assembled in the current version of the genome (Additional file 2: Table S6).

The reference genome of *O. melastigma* was annotated with 25,699 protein-coding genes (avg.exon/coding genes: 8.89) using transcriptome sequencing data from five tissues, combined with ab initio prediction and homology-based approaches. This number is comparable to those found in other vertebrate genomes [40–43] (Table 2). Furthermore, we were able to generate functional assignments for 99.2% of the marine medaka genes from at least one of the public protein databases (Additional file 2: Table S7). The predicted noncoding RNA genes in the *O. melastigma* genome consisted of 926 miRNA, 1916 tRNA, 2474 lncRNA, 825 rRNA and 295 snRNA genes (Additional file 2: Table S8).

**Table 2:** General statistics for the genomes used by the homolog-based method

Species	Number	Average transcript length (bp)	Average CDS length (bp)	Average exons per gene	Average exon length (bp)	Average intron length (bp)
Dre	25,619	25,207.59	1642.64	9.42	174.39	2798.97
Gac	20,787	8451.06	1548.67	10.40	148.94	734.44
Gmo	20,095	15,245.21	1459.03	12.72	114.67	1175.90
Ola	19,699	12,145.58	1515.82	10.25	147.82	1148.61
Ome	25,699	14,538.33	1484.55	8.89	167.04	1655.11
Oni	21,437	14,903.11	1714.22	10.90	157.25	1332.07
Tni	19,602	6066.17	1516.59	10.52	144.20	478.02
Tru	18,523	7492.75	1693.53	11.10	152.61	574.33
Xma	20,379	13,751.42	1643.24	10.69	153.77	1250.06

Note: *Takifugu rubripes* (Tru), *Ctenopharyngodon idellus* (Cid), *Cyprinus carpio* (Cca), *Danio rerio* (Dre), *Sinocyclocheilus graham* (Sga), *Ictalurus punctatus* (Ipu), *Homo sapiens* (Hom) and *Mus musculus* (Mmu)

### Improvements in genome assembly over the previous version

This new genome assembly of *O. melastigma* significantly improved the contiguity in terms of gap-filling and contig sizes. The previously reported marine medaka genome assembly was generated by Illumina reads [25]. Table 3 provides summary statistics for the comparison between previous *O. melastigma* genome assembly and our new assembly. The total length of the new genome assembly is 844 Mb compared to 779 Mb of the previous genome assembly. However, the scaffold N50 (1.71 Mb) of the new genome assembly is shorter than the scaffold N50 (23.73 Mb) of the previous genome assembly. But the new genome assembly contains only 1331 gaps with the length of 8.76 Mb (1.04% of the genome), which is considerably lesser than the previous assembly (51,440 gaps with a total length of 41.24 Mb, 5.29% of the genome). Compared with the previous genome assembly, this assembly represents a considerable decrease in assembly fragmentation (59,791 versus 2588 contigs). We achieved a 25-fold improvement over the previous *O. melastigma* genome assembly (708Kb vs 29Kb) using N50 contig length as a metric. (Additional file 1: Fig. S1). Furthermore, the length distribution of gaps indicated that the previous assembly has many big gaps in addition to thousands of small gaps (Additional file 1: Fig. S2).

**Table 3:** Comparison of previous and new *O. melastigma* assemblies on genomic sequences level

Assembly	Previous assembly		New assembly	
	Contig	Scaffold	Contig	Scaffold
Total size (bp)	738,232,102	779,469,774	835,406,597	844,166,318
Number	59,792	8603	2588	1257
Longest (bp)	268,000	37,948,421	5,175,882	8,672,543
Mean (bp)	12,346	90,604	322,800	671,572
N50	28,594	23,737,187	707,795	1,709,016
%N	0	5.29	0	1.04
Number of gaps	0	51,440	0	1331
Total gap size (bp)	0	41,237,672	0	8,759,721

**Fig. 1:**
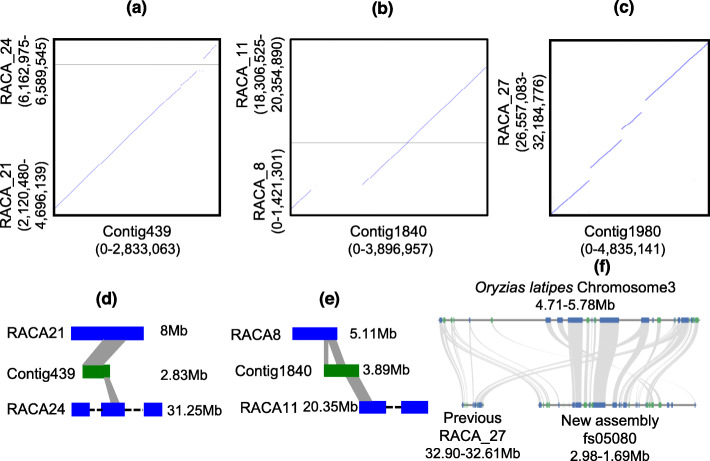
Examples of comparisons between our de novo contigs and the previous assembly. **a**, **d** A dotplot and sketch map between our de novo contig439 and previous assembly, each dot indicates a region of the close similarity between them. Two chromosomes (RACA_21, RACA_24) of the previous assembly mapped to this contig. **b**, **e** A dotplot and sketch map between our de novo contig1840 and previous assembly. Two chromosomes (RACA81, RACA_11) of the previous assembly mapped to this contig. **c** A dotplot between our de novo contig1980 and previous assembly. **f** One local syntenic block among Japanese medaka, previous assembly and our assembly There are some conserved orthologous genes lacked in the previous assembly (Note: the dotted line in d and e means it is a long chromosome)

Previously published *O. melastigma* genome assembly was generated by reference-assisted chromosome assembly (RACA) [44] which ordered sequences generated by Illumina short reads and assemble into chromosomal fragments based on information from closely related species and out-groups. We compared previously assembled chromosomes to our de novo contigs to detect the differences in two assemblies. Both assemblies showed a high mapping rate (94%), but we identified several misassembled regions in the previous genome of *O. melastigma* (Fig. 1). For example, two regions of different chromosomes (RACA_21 and RACA_24) in the previous assembly mapped to contig439 of the current genome assembly (Fig. 1a, d). To validate the accuracy of our contigs, we mapped the PacBio long reads back to our de novo contigs. The read depth of the region around the breakpoint of contig439 showed at least 39 mapping reads which spanned the breakpoint, suggesting this region of contig439 is continuous (Fig. 1a, d; Additional file 1: Fig. S3). The similar phenomenon happened to contig1840 and contig1980 (Fig. 1b, c, e; Additional file 1: Fig. S4; Additional file 1: Fig. S5). Interestingly, the last fragment of RACA_8 and the starting fragment of RACA_11 in the previous assembly is mapped to two distinct regions of contig1840 in the new assembly. These two regions are continuous in contig1840 supported by high mapping rate of PacBio long reads (Additional file 1: Fig. S4). These results indicated that the previous genome misassembled the two pseudo-chromosome RACA_8 and RACA_11. They may belong to one chromosome in *O. melastigma*. Additionally, one fragment (with the length of 5,627,693 bp) at RACA_27 from the previous assembly mapped to contig1980 in the new assembly (with a size of 4,835,141 bp), of which only ~ 4.17 Mb were mapped. Moreover, 77 kb unsequenced gaps in RACA_27 were filled in the new assembly, suggesting that long-read sequencing filled the gaps in the previous assembly (Fig. 1c; Additional file 1: Fig. S5).

**Fig. 2:**
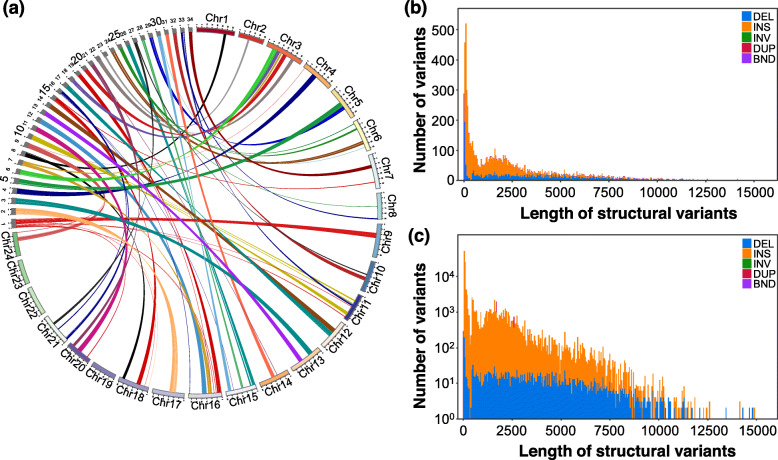
**a** The synteny plot is showing the pairwise synteny comparison between the Japanese medaka chromosomes and the 34 scaffolds of the new version of marine medaka. Labels with Chr1 to Chr24 represents the chromosome of Japanese medaka, while labels with 1 to 34 represent the scaffolds of marine medaka. **b**, **c** Length profile of SV calls. The top right panel has SVs up to 15 kb binned per 50 bp; the bottom right panel, up to 15 kb binned per 50 bp with a log-transformed number of variants

To further illustrate the genome-wide synteny, the syntenic blocks among Japanese medaka, previous and new assemblies of marine medaka were analyzed. A total of 18,724 syntenic gene pairs found between new and previous assemblies. Comparison with Japanese medaka showed that many conserved syntenic genes lacked in the previous genome assembly but located in the new assembly. For example, the synteny was observed between new assembly and Japanese medaka but not between Japanese medaka and previous assembly (Fig. 1f). Furthermore, there are also some rearrangements between Japanese medaka and marine medaka genome even they are closely related species (Fig. 2). A total of 8317 structural variations (SVs) were identified after mapping our long-reads to Japanese medaka. The 5944 SVs overlapped with genes, and 1564 were located in coding sequences (Fig. 2b, c).

**Fig. 3:**
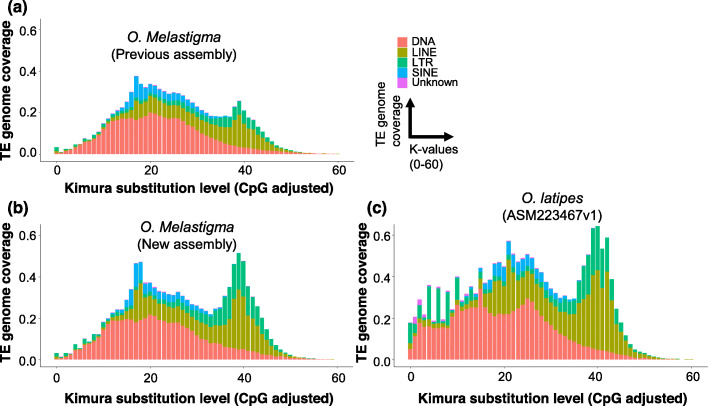
Landscapes of repeat sequence among three assemblies (**a**) previous *O.melastigma* assembly. **b** new version of *O.melastigma* assembly. **c** *O.latipes*. Kimura distance-based copy divergence analysis of transposable elements in *O. latipes*, previous *O. melastigma* and current assembly (Kimura substitution level – CpG adjusted). The graphs represent genome coverage (y-axis) for each type of TEs (DNA transposons, SINE, LINE, LTR retrotransposons and Unknown) in the different genomes analyzed, clustered according to Kimura distances to their corresponding consensus sequence (x-axis, *K*-value from 0 to 60). Copies clustering on the left of the graph do not diverge very much from the consensus sequence of the element and potentially correspond to recent copies, while sequences on the right might correspond to ancient/degenerated copies

The most striking differences between the two versions of *O. melastigma* genome assembly was present within the highly repeated regions. The total size of predicted repetitive elements in the new assembly of *O. melastigma* was 326.6 Mb, accounting for 38.69% of the total genome size, which was higher than the previous assemblies of *O. melastigma* and *O. latipes* (33.67%, 262.5 Mb and 37.84%, 277.8 Mb, respectively, Additional file 2: Table S9). Similarly, long terminal repeat retrotransposons (LTR-RT) (10.5%, 88.67 Mb) and long interspersed nuclear elements (LINE) (15.71%, 132.64 Mb) were also more abundant in the new assembly of *O. melastigma* (Additional file 2: Table S9) than the previous assemblies. Figure 3 demonstrated the repeat elements of new and previously published assemblies of *O. melastigma* and *O. latipes*. Both versions of *O. melastigma* showed similar trends for each type of TEs, except for one major intermediate burst of transposition mainly involving DNA, LINE and SINE, in addition to one significant ancient expansion of LTR and LINE (Fig. 3a, c). The number of repetitive elements increased at almost all divergence levels (Additional file 2: Table S9), with most at higher divergences, especially for the LTR (long terminal repeat) (Additional file 1: Fig. S6). Examining the length distribution of LTR in these two assemblies (Fig. 3a, b), we found more and longer LTRs in the new assembly, suggesting that using single-molecule sequencing reads can overcome the limitations of short-read sequencing by producing long reads which span the repetitive genomic regions. The previous assembly has almost the same size of repetitive elements with Japanese medaka, but with a lower proportion (Additional file 2: Table S9). Overall, TEs are better assembled in the new assembly (Additional file 2: Table S9). Compared with Japanese medaka, TEs in the *O. melastigma* showed less recent activities (Fig. 3).

### Genomic comparison between *Oryzias melastigma* and other vertebrates

Specifying the origin of *O. melastigma* is very important to illustrate the evolution and function of a genome. Mainly, clusters of homologous gene pairs are evidence of candidate homologous regions in distantly related genomes. Demonstrating the statistical significance of such ‘gene clusters’ is an essential component of comparative genomic analyses. A total of 25,595 genes of *O. melastigma* have homologs in other vertebrates and classified into 25,227 orthogroups, 391 of those are single-copy gene families with one-to-one correspondence in different genomes. The distribution of gene family types in each species is shown in Additional file 1: Fig. S7. The trends of gene family types of each species are almost the same except the *Cyprinus carpio* and *Salmo salar* which have more genes. In total, we found 3975 orthogroups shared by all species.

Compared with *Oryzias latipes*, *Nothobranchius furzeri* and *Xiphophorus maculatus*, we found 134 gene families (including 301 genes) unique to *O. melastigma*, which include gene *Hipk1* (Homeodomain-interacting protein kinase 1), apoptosis regulator BAX, *pvrl4*, *Hdlbp*, *CNGB3*, *Fam19a5*, *pycard* and *Clcc1*, etc. Gene Ontology (GO) annotation showed that the unique genes were significantly enriched in functional categories of biological processes, such as apoptotic process (14 genes, Adj. *P*-value = 8.82e-06), programmed cell death (14 genes, Adj. *P*-value = 8.82e-06), cell death (14 genes, Adj. *P*-value = 8.82e-06), death (14 genes, Adj. *P*-value = 8.82e-06), regulation of apoptotic process (12 genes, Adj. *P*-value = 1.36e-05), regulation of cell death (12 genes, Adj. *P*-value = 1.36e-05) and regulation of programmed cell death (12 genes, Adj. *P*-value = 1.36e-05) (Additional file 2: Table S10).

### Phylogenetic relationships

**Fig. 4:**
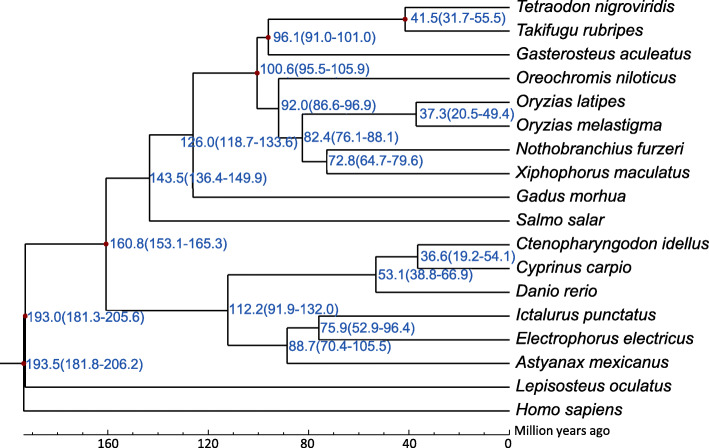
Phylogenetic tree of *O. melastigma* and related species. Estimates of divergence times (millions of years) calculated from the rate of sequence similarity are indicated at each node. Note: The numbers on the nodes represent the divergence times from present (million years ago, Mya), and the numbers in brackets represent the confidence interval values

The availability of genomic dataset improves the capability to precisely examine the evolutionary history and phylogeny of marine medaka. We clustered the *O. melastigma* gene models with the genes from 17 other vertebrate genomes and used 391 single-copy gene families with one-to-one correspondence in the different genomes to reconstruct a high-confidence phylogenetic tree and estimate the divergence times with four calibration points (Fig. 4). As a species of the genus *Oryzias*, *O. melastigma* had the closest relationship with *O. latipes*. According to the TimeTree database, the estimated divergence time between *O. latipes* and *O. melastigma* was around 37.3 million years ago. The relationship among other vertebrate genomes is also in agreement with previous estimates [45, 46].

### Evolutionary adaptation of marine medaka

**Fig. 5:**
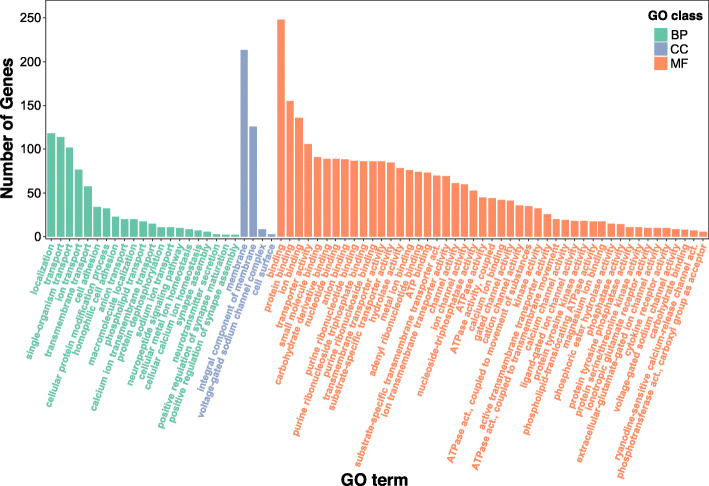
Gene Ontology (GO) enrichment results of expanded gene families (Adjusted *P-value* < 0.05). The horizontal axis displays the detailed GO annotation corresponding to each functional type (biological processes, cellular components and molecular functions). The vertical axis displays the number of expanded genes

Conspicuous expansion or contraction in the size of special gene families is usually connected with the adaptive divergence of closely related species [47, 48]. Based on the result of gene cluster analysis, we undertook a computational analysis of gene family sizes to study gene family expansion and contraction among *O. melastigma* and related species (Additional file 1: Fig. S8). The result showed that there were 25,223 gene families inferred to be present in the most recent common ancestor (MRCA) of mammals. By comparing with the ancestor of *O. melastigma* and *O. latipes*, we found a total of 44 gene families that are significantly (*P*-value< 0.05) expanded in *O. melastigma* and 46 gene families that are significantly contracted (Additional file 2: Table S11). Based on Kyoto Encyclopedia of Genes and Genomes (KEGG) of genes from most of these expanded gene families, we found high enrichment of KEGG pathways includes: calcium signaling pathway (Adj. *P*-value = 3.48e-18), ABC transporters (Adj. *P*-value = 5.78e-16), cell adhesion molecules (CAMs)(Adj. *P*-value = 5.10e-13), circadian entrainment (Adj. *P*-value = 1.18e-12), long-term potentiation (Adj. *P*-value = 2.49e-09), systemic lupus erythematosus (Adj. *P*-value = 1.10e-07) and so on (Table 4; Additional file 2: Table S12). Furthermore, for significantly expanded gene families, we conducted the gene ontology (GO) enrichment analyses and found enrichment for GO terms such as ‘ATPase activity’ (Adj. *P*-value = 1.15e-31), ‘transmembrane transport’ (Adj. *P*-value = 2.27e-25), ‘ATPase activity, coupled to movement of substances’ (Adj. *P*-value = 1.74e-23), ‘phospholipid-translocating ATPase activity’ (Adj. *P*-value = 3.38e-23), ‘phospholipid transport’ (Adj. *P*-value = 3.38e-23), ‘calcium ion binding’ (Adj. *P*-value = 3.99e-13), ‘ion channel activity’ (Adj. *P*-value =1.33e-08), ‘voltage-gated sodium channel activity’ (Adj. *P*-value =6.91e-08), ‘ion transport’ (Adj. *P*-value =4.37e-06), etc. (Additional file 2: Table S13; Fig. 5).

**Table 4:** Functional annotation of the most significantly expanded and contracted gene families in *O. melastigma*

Gene families	KEGG terms	Input no.	Background no.	***P***-value
Expanded gene families	Calcium signaling pathway	33	470	3.48E-18
	ABC transporters	18	66	5.78E-16
	Cell adhesion molecules (CAMs)	24	345	5.10E-13
	Circadian entrainment	26	251	1.18E-12
	Long-term potentiation	18	162	2.49E-09
	Systemic lupus erythematosus	14	118	1.10E-07
	Cocaine addiction	12	100	9.23E-07
	Nicotine addiction	12	100	9.23E-07
	Amyotrophic lateral sclerosis (ALS)	12	106	1.60E-06
	Alzheimer’s disease	19	293	3.09E-06
	Dilated cardiomyopathy	16	229	9.50E-06
	Measles	15	216	2.08E-05
	Amphetamine addiction	12	150	4.78E-05
	Asthma	7	45	5.37E-05
	*Staphylococcus aureus* infection	10	107	6.44E-05
	Axon guidance	18	427	0.000275
	Intestinal immune network for IgA production	7	69	0.000764
	Hypertrophic cardiomyopathy (HCM)	12	205	0.000774
	Viral myocarditis	10	152	0.001042
	Allograft rejection	7	80	0.001658
	Alcoholism	12	227	0.001718
	Autoimmune thyroid disease	7	92	0.003543
	Glutamatergic synapse	12	266	0.006097
Contracted gene families	Tight junction	12	306	4.30E-10
	Systemic lupus erythematosus	5	118	0.000282
	Ascorbate and aldarate metabolism	3	30	0.000866
	Axon guidance	7	427	0.000959
	Drug metabolism - cytochrome P450	3	37	0.000959
	Porphyrin and chlorophyll metabolism	3	39	0.000959
	Drug metabolism - other enzymes	3	42	0.000964
	Metabolism of xenobiotics by cytochrome P450	3	43	0.000964
	Steroid hormone biosynthesis	3	50	0.001068
	Alcoholism	5	227	0.001068
	Pentose and glucuronate interconversions	3	51	0.001068
	Chemical carcinogenesis	3	51	0.001068
	Retinol metabolism	3	68	0.002299
	Starch and sucrose metabolism	3	75	0.002839

The evolutionary adaptations of *O. melastigma* populations could have been accompanied by dramatic changes in the environment, such as oil contamination, heavy metals, temperature variation, salinity and pH of seawater. These changes resulted in powerful selective pressures for new genotypes that were better suited in harsh environments. So, signals of very recent positive selection were identified, which provide information about the genetic adaptations of *O. melastigma* to local environmental conditions. We found 274 highly significant (Adj. *P*-value < 0.005) positively selected genes (PSGs) in *O. melastigma* through a likelihood ratio test (Additional file 2: Table S14). KEGG and GO annotations results showed the involvement of PSGs in the ‘DNA damage repair’ functional category (e.g., *DDB2*, *RAD23B*, *CHST11*, *MRE11* and *XRCC3*) (Additional file 2: Table S15; Additional file 2: Table S16). Interestingly, *RAD23B* was located in ‘nucleotide excision repair’, *DDB2* in ‘nucleotide excision repair’ and ‘p53 signaling pathway’, *CHST11* in ‘biosynthesis of amino acids’, *MRE11* and *XRCC3* were located in ‘homologous recombination’ functional categories. Furthermore, many of the PSGs (e.g., *PIGB*, *VAMP8*, *RFWD2*, *RPE* and *MYADM*) in the *O. melastigma* genome were enriched in the transporter activities (including ‘peptide transporter activity’, ‘peptide transport’, ‘amide transport’, ‘nitrogen compound transport’, ‘ion homeostasis’ and ‘cation homeostasis’).

## Discussion

This genome sequencing projects of *O. melastigma* aimed to generate a high-quality reference assembly that can serve as a foundation for various downstream analyses, such as gene finding, variant identification, and comparative and functional assays. Several commonly used second-generation genome sequencing approaches provide gigabases of data [49, 50]. Although these approaches offer higher sequencing depth per sample, the short-read sequencing approach limits the assembly of longer contigs, especially when sequencing the complex heterozygous genomes. Other limitations include that repetitive genomic region in complex genomes is often poorly assembled with short-read sequencing approach [50, 51]. Therefore, in current study, PacBio sequencing provides longer reads which make it easy to sequence through extended repetitive regions and to detect large-scale mutations [52]. The sequence data from both PacBio and 10X Genomics linked-read sequencing can be used to extend contigs and/or fill in the gaps between neighbouring contigs [53]. So, this de novo genome assembly of *O. melastigma* was done by combining the above mentioned methods to cover the regions that would be problematic for short-read sequencing methods. This combined approach has been illustrated to be suitable for highly heterozygous genomes [28, 30, 54, 55].

The advent of PacBio sequencing resulted in the first long-read based genome assembly of *O. melastigma*. We compared the quality of our new assembly with the previous assembly of *O. melastigma* based on the short Illumina sequencing reads [25]. The contig N50 size of our new assembly was substantially higher than the previous genome assembly of *O. melastigma*, highlighting the exclusive benefits of long-read sequencing methods in assembling complex genomes. Similarly, Weisenfeld et al., (2017) have also reported that using long-read genome sequencing enables a genome assembly to achieve both high sequence contiguity as well as high scaffold contiguity. Besides, the new genome assembly of *O. melastigma* revealed numerous errors and filled gaps within and surrounding many genomic regions in the previous assembly. These errors are not limited to intergenic repetitive DNA regions known to be hard to assemble with short reads [56, 57], but also located within functional regions of genes. For example, we compared the syntenic blocks among previous assembly chromosomes with our de novo contigs. The results showed several misassembles of pseudo-chromosomes and several conserved orthologous genes were lacked in the previous assembly. Moreover, we compared the distribution of long terminal repeat retrotransposon (LTR-RT) copies among new assembly and previous assemblies [25, 41]. Our genome assembly can detect more and longer LTR-RT compared with the previous assemblies, demonstrating that single-molecule sequencing of complex genomes can overcome the complications of the short-read sequencing by producing the longer reads spanning repetitive genomic regions.

The extreme conditions in the polluted sea-water may influence the osmoregulation, growth and developmental processes in *O. melastigma*, possibly causing DNA, RNA, and protein damages. Notably, we were able to identify several unique genes, expanded gene families, and genes that underwent positive selection possibly linked to evolutionary adaptations of *O. melastigma*. GO and KEGG functional assays revealed that the molecular functions of those genes are involved in; i) homologous recombination (HR) and nucleotide excision repair pathways, which are essential mechanism to recognize and accurately repair the bulky DNA double-strand breaks (DSBs) [58], ii) an important p53 pathway which is a critical factor that helps to conserve the stability of the genome by preventing mutations caused by cellular stress or DNA damage [59], and iii) the transmembrane transportation and ion transmembrane transportation processes to maintain cell homeostasis for continuous and proper functioning of the cell. For example, in this study we identified *DDB2,* a gene in the nucleotide excision repair and p53 pathways, was positively selected and was known to perform critical functions related to DNA damage repair (such as chromatin remodeling, cell cycle arrest and homologous recombinational) caused by the ionizing radiation or carcinogenic benzo(a) pyrene metabolite [60–63]. Similarly, we found nine *ABCC* expanded genes enriched in ATP-binding cassette (ABC) transporter pathways, where they may regulate the transportation of diverse substances (such as drugs, sterols, ions, sugars, peptides, lipids and proteins) [64, 65]. These results and pieces of evidence from previous studies suggested that expansion/duplication and subsequent positive selection of genes are essential mechanisms for evolutionary adaptation in animals.

Taken together, our improved *de-novo* genome assembly of *O. melastigma* (with more complete and accurately assembled genes of interests) will serve as an ideal reference for future studies based on genome evolution. Moreover, comparative genomic results and functional annotation of expanded and positively selected genes will provide a solid foundation for further investigation of molecular responses of *O. melastigma* to marine environmental stressors.

## Conclusions

Marine medaka is considered as a model organism to illustrate the toxicological impacts on the marine ecosystem. In this study, we demonstrated the deployment of long-read sequence technology to generate high-quality, accurate and near to complete draft genome of marine medaka. Comparison with the previous published marine medaka genome based on second-generation sequencing platform and assembled with the assistant of related species indicates that our long-read assembly provides superior performance in terms of contig length, gene contents, gaps filling and repeat sequence detection. Our assembly has a length of 844Mbp, which corresponds to 98.75% of the estimated size of the genome. The results of our study highlighted that the use of single-molecule sequencing reads could overcome the limitations of short-read sequencing. Using this version of the genome, we identified gene families that underwent significant expansion and genes showed the signature of positive selection are enriched in DNA damage repair and cellular transportation of diverse substances pathways, which reflect the evolutionary adaptations of *O. melastigma*. The highly contiguous marine medaka genome and comparative genomic analyses will increase our understanding of mechanisms of its extraordinary adaptation capability and significantly accelerate researches in marine ecotoxicology.

## Methods

### Sample preparation and sequencing

All animal procedures were carried out in strict compliance with the National Institute of Health Guidelines for the Care and Use of Laboratory Animals and were approved by the animal welfare and ethics committee of Xiamen University. The marine medaka was provided by the State Key Laboratory of Marine Environmental Science, Xiamen University, the State Key Laboratory in Marine Pollution, City University of Hong Kong. Our laboratory established a self-propagating population of marine medaka (bigg-433). The total of 8 mature (five-month-old) male marine medaka were collected, and instantly anaesthetised with dry ice bath for 10s. Muscles of two deeply anaesthetized mature male marine medaka were dissected, and their DNA was extracted for genome sequencing. We used DNA from one mature male of marine medaka for PacBio sequencing, and another mature sibling male for Illumina sequencing. DNA from one fish was insufficient to construct all libraries for sequencing. We also dissected the brain, heart, gill, gonad, muscle from the other six male marine medaka, and extracted the RNA for RNA sequencing.

### PacBio library construction and sequencing

Genomic DNA was isolated from the muscles of marine medaka. The qualified genomic DNA was fragmented randomly by ultrasonication (Covaris) and concentrated using the AMPure PB magnetic beads. Then, followed by PacBio SMRTbell 20 kb Library Preparation procedures to construct a 20-kb insert size library. Finally, we sequenced the DNA library on the PacBio Sequel platform, yielding about 68.61 Gb pacbio data (mean read length ≥ 7.9 Kb) (Additional file 2: Table S1). Subreads were filtered with the default parameters. We used falcon [66] to do self-correction and assembly for pacbio data. Then we corrected and polished the assembly to generate high-quality consensus sequences efficiently by Arrow in SmrtLink v5.0.1 [67].

### 10X genomics library construction, sequencing and extending scaffolds

10X Genomics provides an integrated microfluidics-based platform for generating linked reads and customized software for their analysis [53, 68]. A 10X Genomics library was constructed according to manufacturer’s instructions, and a lane of Illumina HiSeqX ten 150 bp paired-end reads was generated with a coverage of about 117.85X. We used BWA mem [36] to align the 10X Genomics linked-reads to consensus sequences gained by PacBio using default settings. Then, we used fragScaff [69] for scaffolding.

A standard protocol to correct PacBio long reads was adopted as a second-generation sequencing platform (like Illumina) to assemble a genome with an error rate of less than 1%. To achieve this goal, one paired-end Illumina sequence library was constructed with an insert size of 350 bp, and sequencing was carried out on the Illumina HiSeqX ten platform according to the manufacturer’s instructions; 146.91 Gb (172X coverage) sequencing data were produced. The following criteria filtered raw sequence data generated by the Illumina platform: filtered reads with adapters, filtered reads with N bases more than 10%, and filtered reads with more than 20% of low-quality bases (≤ 5). We used BWA [36] to align all the short clean data to the assembly, then used Pilon [70] with default settings to correct assembled errors.

### Repeat prediction

ab initio repeat annotation of marine medaka genome was first carried out by successively using RepeatScout [51], TRF (Tandem Repeats Finder) [71] and LTR_FINDER [72]. The marine medaka repeat library was finally constructed by RepeatMasker [73] through the combined database between ab initio repeat library and the Repbase transposable element library [74]. The identification and classification of genomic repeats were conducted by Piler [75].

### Gene and non-coding RNA prediction

EVidenceModeler [76] was used to generate a nonredundant and complete gene set based on ab initio predictions from AUGUSTUS [77], GlimmerHMM [78], SNAP [79], GeneID [80] and Genscan [81], homology annotation with the universal single-copy genes from related species (*Danio rerio*, *Oryzias latipes*, *Xiphophorus maculatus*, *Tetraodon nigroviridis*, *Takifugu rubripes*, *Gasterosteus aculeatus*, *Gadus morhua*, and *Oreochromis niloticus*) (Additional file 2: Table S17) and RNA-seq alignment data. For RNA-seq data derived from the brain, heart, gill, gonad, muscle, we removed adaptor sequences and filtered low-quality reads by using Trimmomatic [82]. The clean reads were de novo assembled and annotated with the Trinity [83], PASA program [84] and Cufflinks [85] after mapping to the new assembly by tophat [86]. Then combined RNA-seq prediction to correct the EVidenceModeler result by PASA and add UTR and alternative splicing information. These results were integrated into a final set of protein-coding genes for annotation.

We then generated functional assignments of the marine medaka genes with BLAST [87] and GeneWise [88] by aligning their protein-coding regions to sequences in public protein databases, including SwissProt [89], NCBI non-redundant protein database*,* Pfam [90], Gene Ontology [91], KEGG [92] and InterPro [93].

The rRNA fragments were predicted by aligning the rRNA sequences of related species because of high conservation. The tRNA genes were searched by tRNAscan-SE [94]. Additionally, miRNA and snRNA were identified by using INFERNAL [95] to search from the Rfam database [96]. CPC2 [97] and CPAT [98] identified the lncRNAs. Transcripts encoding ORFs longer than 100 amino acids were filtered, and the remaining transcripts were further screened by BLASTX (e-value <1e-10) against the SwissProt and Nr database.

### Gene family identification

We downloaded genome and annotation data of *Nothobranchius furzeri*, *Salmo salar*, *Cyprinus carpio*, *Ictalurus punctatus*, *Ctenopharyngodon idellus*, *Oryzias latipes*, *Xiphophorus maculatus*, *Oreochromis niloticus*, *Takifugu rubripes*, *Tetraodon nigroviridis*, *Gasterosteus aculeatus*, *Gadus morhua*, *Danio rerio*, *Astyanax mexicanus*, *Lepisosteus oculatus*, *Homo sapiens* and *Electrophorus electricus* (see Additional file 2: Table S17). We chose the longest transcript to represent each gene and removed gene models encode less than 30 amino acids. The similarities among proteins were obtained by blastp [87] with an *E*-value cutoff of 1e-5. Gene family clustering was conducted using OrthoMCL [99] based on the set of predicted genes of *O. melastigma* and the protein sets of the above 17 species. This analysis yielded 25,227 gene families.

### Phylogenetic tree construction and phylogenomic dating

A phylogenetic tree was constructed based on a concatenated sequence alignment of 391 single-copy gene families from marine medaka and the 17 other related species. These single-copy gene families were firstly aligned by MUSCLE [100], then concatenated to a super alignment matrix. In the end, ML phylogenic tree was constructed using RaxML [101]. PAML MCMCTree [102] estimated divergence times. The Markov chain Monte Carlo (MCMC) process was run with a sample number of 1,000,000, a sampling frequency of two after a burn-in of 1000 iterations. Other parameters used the default settings of MCMCTree. The following constraints were used for time calibrations: (i) the *Tetraodon nigroviridis* and *Gasterosteus aculeatus* divergence time [149–166 million years ago (Mya)]; (ii) the *Oryzias latipes* and *Gasterosteus aculeatus* divergence time (97–151 Mya); (iii) the *Lepisosteus oculatus* and other 16 fish species divergence time (291–338 Mya); and (iv) the *Homo sapiens* and other 17 species divergence time (416-422Mya). Estimation of gene family expansion and contraction were done using CAFÉ [103].

### Detecting positive selection in the genome

Sequence alignments were conducted using the MUSCLE [100] tool for single-copy gene families. Both nonsynonymous (dN) and synonymous substitution rates (dS) and dN/dS ratio (ω) of every lineage were estimated using the branch-site model analysis with codeml program in PAML [104–106]. Based on a maximum likelihood ratio test (LRT), we identified genes under positive selection in marine medaka. These genes were identified as positively selected according to the chi-squared test (*P*-value < 0.01, FDR < 0.05, df = 1), and containing amino acid sites that were selected with a Bayes probability higher than 95%.

### Calling of variants

PacBio subreads were aligned to new assembly (contig level) using NGMLR (v0.2.7) [107] to generate the BAM file. The BAM file was sorted by SAMtools [37], then used as the input of Sniffles (version 1.0.11) [107] to identify structural variant. Jcvi [108] was used to detect the syntenic blocks.

SAMtools package [37] was used to perform SNP calling based on bam file (generated earlier, the same Illumina data used to correct assembly errors). Raw vcf files were filtered, and SnpEff [109] software was used to annotate the variable sites.

### Go annotation

Significantly overrepresented GO terms in this study were identified using the topGO [110] package in R programming language, and the FDR correction was applied. Significantly overrepresented GO terms were identified with corrected *P*-values of ≤0.05.

## Supplementary information

**Additional file 1: Figure S1**. Length distribution of gaps in the previous version (left) and new assembly (right). There are 51,440 and 1,331 gaps in the previous version and new assembly. Moreover, the maximum gap length of them was 892,371 bp and 8,013 bp separately. **Figure S2**. Length distribution of contigs in the previous version (A) and new assembly (B). There are 59,791 and 2,589 contigs in previous version (contig N50 28,594 bp) and new assembly (contig N50 707,795 bp. Furthermore, the maximum contig length of them were 268,000 bp and 5,175,882 bp separately. **Figure S3**. The read depth of the region around breakpoint of new *de novo* contig439. Mapping of PacBio long reads to *de novo* contig439 to showed if it is continuous near 2.57Mb of the contig. **Figure S4**. The read depth of the region around breakpoint of new *de novo* contig1840. Mapping of PacBio long reads to *de novo* contig1840 to showed if it is continuous near 2.31Mb of the contig. **Figure S5**. The read depth of the region around breakpoint of new *de novo* contig1980. Mapping of PacBio long reads to *de novo* contig1980 to showed if it is continuous. **Figure S6**. The length distribution of long terminal repeats (LTR) families for new assembly and previous assembly. **Figure S7**. The distribution of gene family types which include single-copy orthologs, multiple-copy orthologs, unique and other orthologs in each species. **Figure S8**. Estimation of gene family expansion and contraction using CAFÉ. Clock calibrated phylogenetic tree showing the number of gene families significantly (*P*-value ≤ 0.01) expanded (green), contracted (red). MRCA: most recent common ancestor.

**Additional file 2: Table S1**. Genomic characteristics statistics of *Oryzias melastigma* (Kmer=17). **Table S2**. Sequencing data used for the *Oryzias melastigma* genome assembly. **Table S3**. The mapping rate and coverage rate of short read sequences. **Table S4**. Statistics of variants calling. **Table S5**. Number of SNP effects by region in the marine medaka genome. **Table S6**. Genome completeness as measured by CEGMA and BUSCO. **Table S7**. Statistical of predicted functional genes in public protein databases. **Table S8**. The number of all kinds of non-coding RNA. **Table S9**. Summary statistics of repeat elements*.* **Table S10**. Significantly over-represented Gene Ontology (GO) terms among *O. melastigma*-specific genes compared with *Oryzias latipes, Nothobranchius furzeri* and *Xiphophorus maculatus*. “X” is the number of *O. melastigma*-specific genes assigned to that GO term. The GO terms with corrected *P-*value bellow 0.05 are selected as significantly enriched groups. **Table S11**. The list of 44 expanded gene families and 46 contracted gene families that appeared unique to *Oryzias melastigma*. **Table S12**. KEGG pathway results of expanded gene families. **Table S13**. GO functional enrichment results for expanded gene families. **Table S14**. Positively selected genes in the *O. melastigma*. **Table S15**. Gene Ontology (GO) enrichment of positively selected genes (PSGs) in the *O. melastigma.* **Table S16**. KEGG pathway descriptions of those positively selected genes in *O. melastigma*, which showed significant *P-*value (0.05). **Table S17**. Species included in the comparative genomics in this study.

## Data Availability

All sequence data that support the findings of this study have been deposited in GenBank with the following accession numbers: WKFB00000000 for whole-genome sequence assembly under BioProject accession PRJNA556761; SRX8937101 for sequences of the 350 bp library, SRX8937099 for those from the 500-700 bp library, SRX8937100 for those from the 20 kb long-read PacBio library; SRX8911616 -to- SRX89116120 for RNA-Seq data set for heart, muscle, gonad, brain and gill transcriptome. The web links corresponding to the genome and annotation datasets for *Nothobranchius furzeri*, *Salmo salar*, *Cyprinus carpio*, *Ictalurus punctatus*, *Ctenopharyngodon idellus*, *Oryzias latipes*, *Xiphophorus maculatus*, *Oreochromis niloticus*, *Takifugu rubripes*, *Tetraodon nigroviridis*, *Gasterosteus aculeatus*, *Gadus morhua*, *Danio rerio*, *Astyanax mexicanus*, *Lepisosteus oculatus*, *Homo sapiens* and *Electrophorus electricus* are listed in Additional file 2: Table S17.

## Abbreviations

ABC: ATP-binding cassette
SMRT sequencing: Single Molecule, Real-Time sequencing
ppt: Parts per thousand
PE: Paired-end
MP: Mate-pair
BWA: Burrows-Wheeler Aligner
SNP: Single-nucleotide polymorphisms
BUSCO: Benchmarking Universal Single-Copy Orthologs
CEGMA: Core Eukaryotic Genes Mapping Approach
RACA: Reference-assisted chromosome assembly
SV: Structural variation
LTR-RT: Long terminal repeat retrotransposon
LINE: Long interspersed nuclear element
SINE: Short interspersed nuclear element
LTR: Long terminal repeat
GO: Gene Ontology
KEGG: Kyoto Encyclopedia of Genes and Genomes
MRCA: most recent common ancestor
CAMs: Cell adhesion molecules
PSG: Positively selected gene
HR: Homologous recombination
DBS: Double-strand break
PASA: Program to Assemble Spliced Alignments
UTR: Untranslated region
PAML: Phylogenetic Analysis by Maximum Likelihood
MCMC: Markov chain Monte Carlo
Mya: Million years ago
FDR: False discovery rate
