# Supplementary material for: Long-read sequencing and de novo genome assembly of marine medaka (Oryzias melastigma)
Source: BMC Genomics. 2020 Sep 16;21:640. doi: 10.1186/s12864-020-07042-7 (PMC7493909; doi:10.1186/s12864-020-07042-7)
Supplement: Supplementary file 1 — Additional file 1: Figure S1. Length distribution of gaps in the previous version (left) and new assembly (right). There are 51,440 and 1,331 gaps in the previous version and new assembly. Moreover, the maximum gap length of them was 892,371 bp and 8,013 bp separately. Figure S2. Length distribution of contigs in the previous version (A) and new assembly (B). There are 59,791 and 2,589 contigs in previous version (contig N50 28,594 bp) and new assembly (contig N50 707,795 bp. Furthermore, the maximum contig length of them were 268,000 bp and 5,175,882 bp separately. Figure S3. The read depth of the region around breakpoint of new de novo contig439. Mapping of PacBio long reads to de novo contig439 to showed if it is continuous near 2.57Mb of the contig. Figure S4. The read depth of the region around breakpoint of new de novo contig1840. Mapping of PacBio long reads to de novo contig1840 to showed if it is continuous near 2.31Mb of the contig. Figure S5. The read depth of the region around breakpoint of new de novo contig1980. Mapping of PacBio long reads to de novo contig1980 to showed if it is continuous. Figure S6. The length distribution of long terminal repeats (LTR) families for new assembly and previous assembly. Figure S7. The distribution of gene family types which include single-copy orthologs, multiple-copy orthologs, unique and other orthologs in each species. Figure S8. Estimation of gene family expansion and contraction using CAFÉ. Clock calibrated phylogenetic tree showing the number of gene families significantly (P-value ≤ 0.01) expanded (green), contracted (red). MRCA: most recent common ancestor. [file 12864_2020_7042_MOESM1_ESM.docx]

***Supplementary Figures***

**Long-read sequencing and *de novo* genome assembly of marine medaka (*Oryzias melastigma*)**

Pingping Liang^1^, Hafiz Sohaib Ahmed Saqib^2^, Xiaomin Ni^1,3^, Yingjia Shen^1, *^

Supplementary figures (8 total)


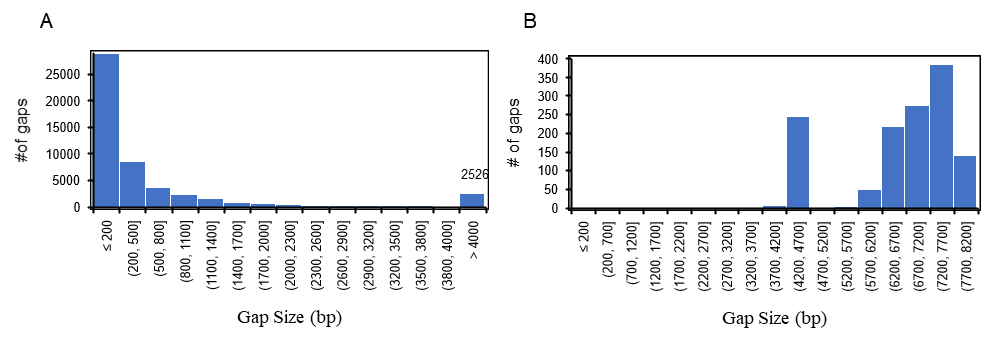


**Figure S1. Length** **distribution of gaps in the previous version (left) and new assembly (right).** There are 51,440 and 1,331 gaps in the previous version and new assembly. Moreover, the maximum gap length of them was 892,371 bp and 8,013 bp separately.


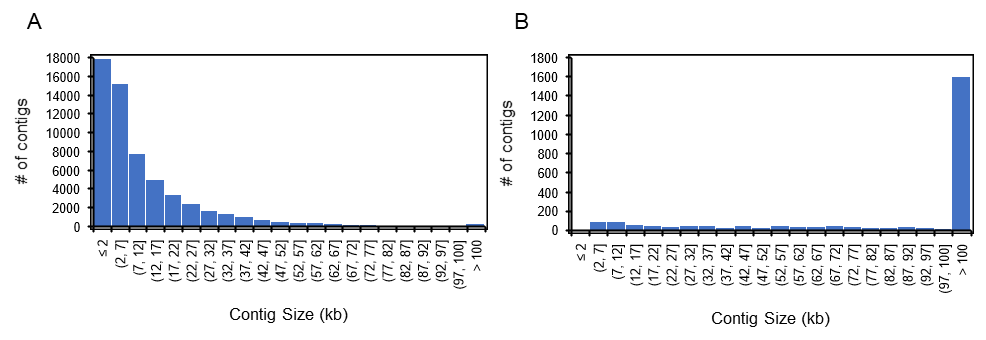


**Figure S2. Length distribution of contigs in the previous version (A) and new assembly (B).** There are 59,791 and 2,589 contigs in previous version (contig N50 28,594 bp) and new assembly (contig N50 707,795 bp. Furthermore, the maximum contig length of them were 268,000 bp and 5,175,882 bp separately.


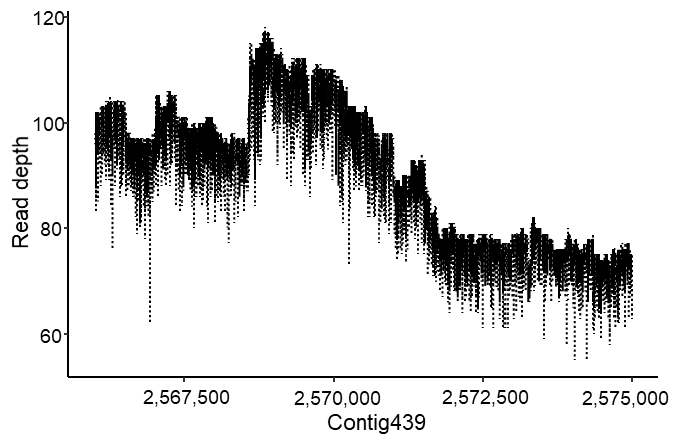


**Figure S3. The read depth of the region around breakpoint of new *de novo* contig439.** Mapping of PacBio long reads to *de novo* contig439 to showed if it is continuous near 2.57Mb of the contig.


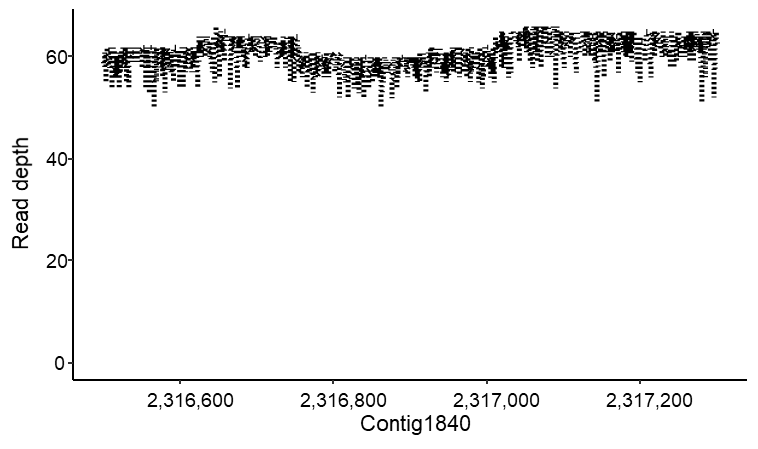


**Figure S4. The read depth of the region around breakpoint of new** *de novo* **contig1840.** Mapping of PacBio long reads to *de novo* contig1840 to showed if it is continuous near 2.31Mb of the contig.


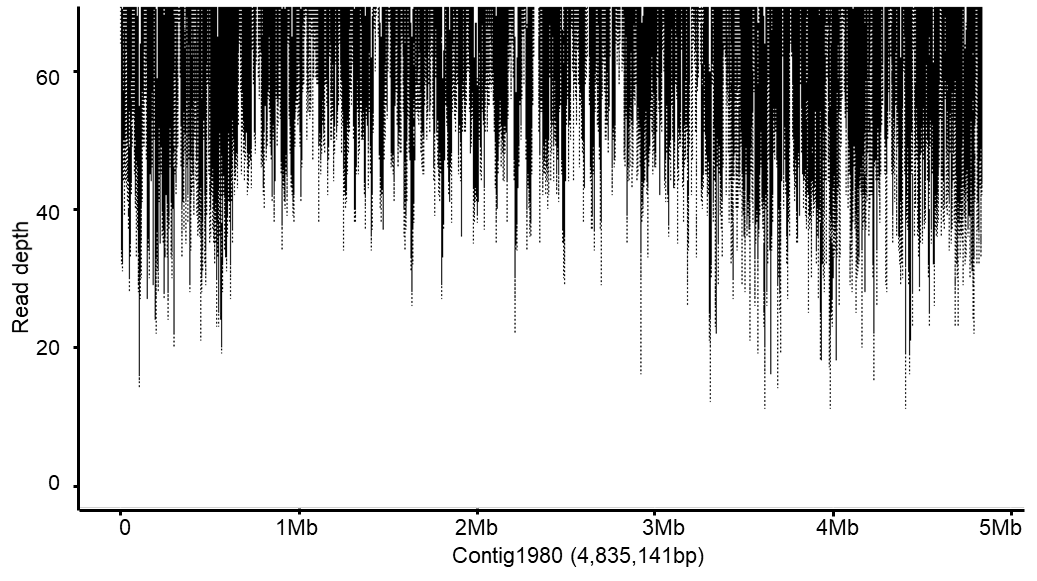


**Figure S5. The read depth of the region around breakpoint of new *de novo* contig1980.** Mapping of PacBio long reads to *de novo* contig1980 to showed if it is continuous.


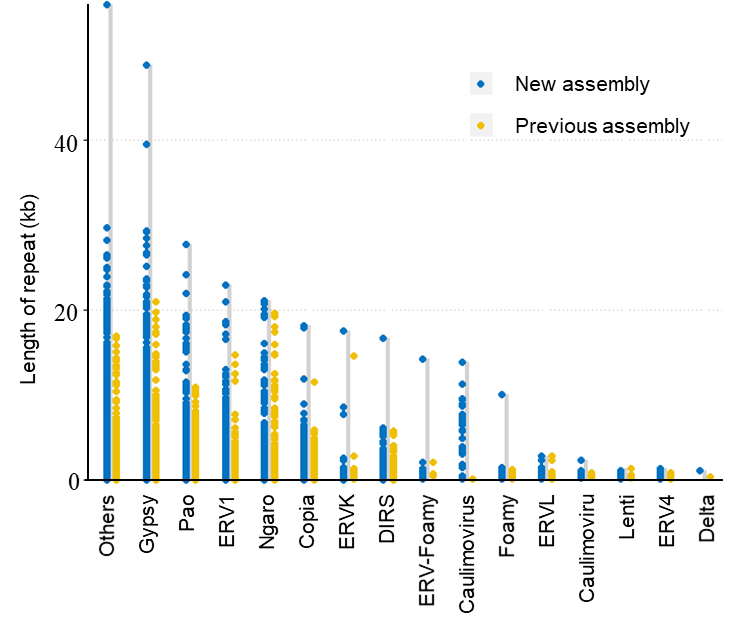


**Figure S6. The length distribution of long terminal repeats (LTR) families for new assembly and previous assembly.**


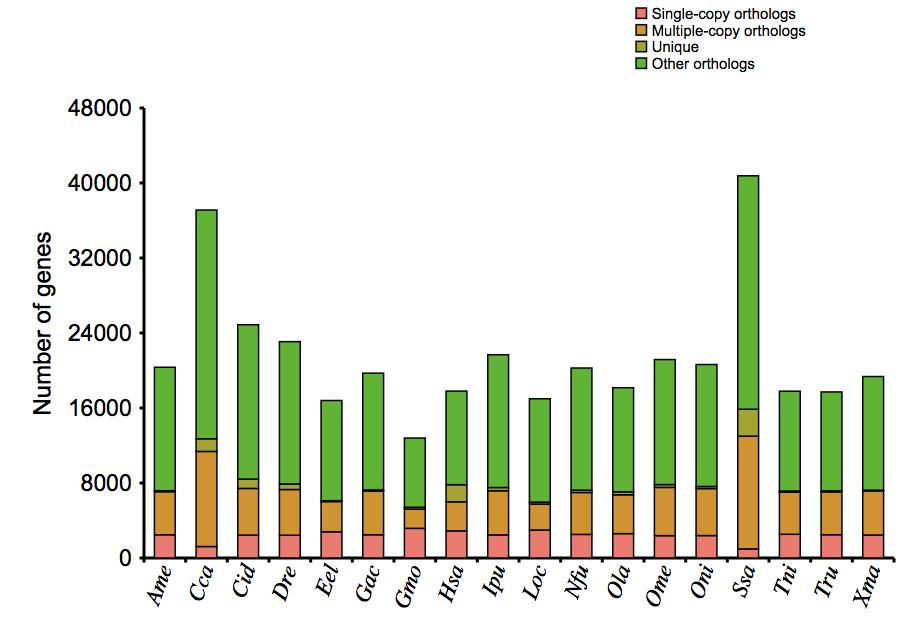


**Figure S7. The distribution of gene family types which include single-copy orthologs, multiple-copy orthologs, unique and other orthologs in each species.**


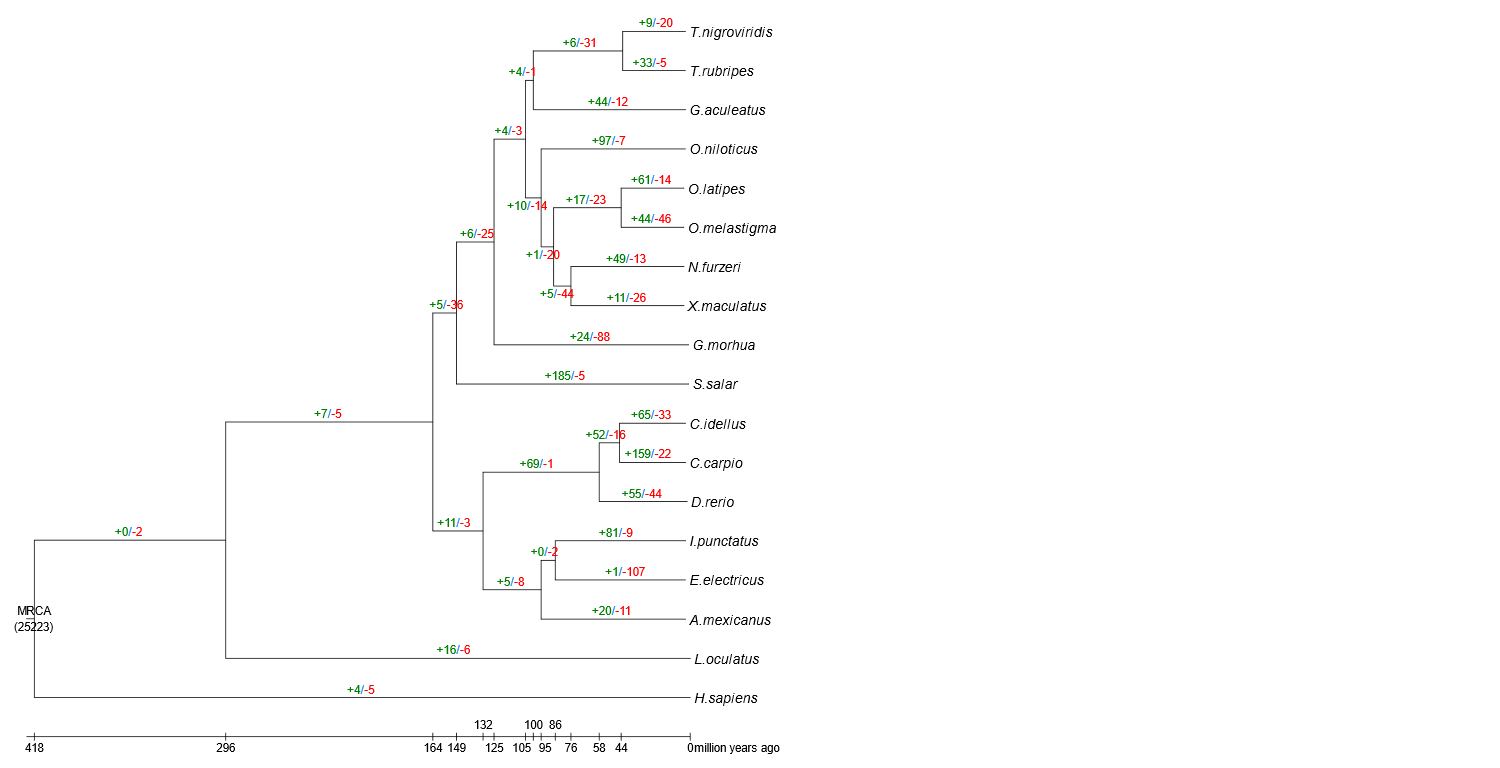


**Figure S8. Estimation of gene family expansion and contraction using CAFÉ.** Clock calibrated phylogenetic tree showing the number of gene families significantly (P-value ≤ 0.01) expanded (green), contracted (red). MRCA: most recent common ancestor.
