# Supplementary material for: Long-read sequencing and de novo genome assembly of marine medaka (Oryzias melastigma)
Source: BMC Genomics. 2020 Sep 16;21:640. doi: 10.1186/s12864-020-07042-7 (PMC7493909; doi:10.1186/s12864-020-07042-7)
Supplement: Supplementary file 2 — Additional file 2: Table S1. Genomic characteristics statistics of Oryzias melastigma (Kmer=17). Table S2. Sequencing data used for the Oryzias melastigma genome assembly. Table S3. The mapping rate and coverage rate of short read sequences. Table S4. Statistics of variants calling. Table S5. Number of SNP effects by region in the marine medaka genome. Table S6. Genome completeness as measured by CEGMA and BUSCO. Table S7. Statistical of predicted functional genes in public protein databases. Table S8. The number of all kinds of non-coding RNA. Table S9. Summary statistics of repeat elements. Table S10. Significantly over-represented Gene Ontology (GO) terms among O. melastigma-specific genes compared with Oryzias latipes, Nothobranchius furzeri and Xiphophorus maculatus. “X” is the number of O. melastigma-specific genes assigned to that GO term. The GO terms with corrected P-value bellow 0.05 are selected as significantly enriched groups. Table S11. The list of 44 expanded gene families and 46 contracted gene families that appeared unique to Oryzias melastigma. Table S12. KEGG pathway results of expanded gene families. Table S13. GO functional enrichment results for expanded gene families. Table S14. Positively selected genes in the O. melastigma. Table S15. Gene Ontology (GO) enrichment of positively selected genes (PSGs) in the O. melastigma. Table S16. KEGG pathway descriptions of those positively selected genes in O. melastigma, which showed significant P-value (0.05). Table S17. Species included in the comparative genomics in this study. [file 12864_2020_7042_MOESM2_ESM.docx]

***Supplementary Tables***

**Long-read sequencing and *de novo* genome assembly of marine medaka (*Oryzias melastigma*)**

Pingping Liang^1^, Hafiz Sohaib Ahmed Saqib^2^, Xiaomin Ni^1,3^, Yingjia Shen^1, *^

Supplementary tables (17 total)

**Table S1. Genomic characteristics statistics of *Oryzias melastigma* (Kmer=17).**

| **K-mer** | **K-mer number** | **K-mer Depth** | **Genome Size（Mbp）** | **Revised Genome Size (Mbp)** | **Heterozygous Ratio (%)** | **Repeat (%)** |
| --- | --- | --- | --- | --- | --- | --- |
| 17 | 110,764,107,076 | 128 | 865.34 | 854.86 | 0.83 | 46.10 |

All 17-mer sequences were extracted from paired-end clean reads that passed quality control (QC) from Next-generation sequencing libraries (350 bp), and the frequency of each 17-mer was calculated and plotted.

**Table S2. Sequencing data used for the *Oryzias melastigma* genome assembly.**

| **Pair-end libraries** | **Insert size** | **Total data (G)** | **Read length (bp)** | **Sequence coverage (X)** |
| --- | --- | --- | --- | --- |
| Illumina reads | 350bp | 146.91 | 150 | 171.84 |
| Pacbio reads | - | 68.61 | - | 80.26 |
| 10X Genomics | - | 100.76 | 150 | 117.85 |
| Total | - | 316.28 | - | 369.96 |

Note: The coverage was calculated based on the estimated genome size (854.86Mb) by the survey.

**Table S3. The mapping rate and coverage rate of short read sequences.**

|  |  | Percentage |
| --- | --- | --- |
| Reads | Mapping rate (%) | 96.19 |
| Genome | Average sequencing depth | 138.21 |
|  | Coverage (%) | 99.15 |
|  | Coverage at least 4X (%) | 98.86 |
|  | Coverage at least 10X (%) | 98.55 |
|  | Coverage at least 20X (%) | 98.19 |

**Table S4. Statistics of variants calling.**

|  | Number | Percentage（%） |
| --- | --- | --- |
| All SNP | 3,785,501 | 0.47 |
| Heterozygosis SNP | 3,756,890 | 0.4664 |
| Homology SNP | 28,611 | 0.0036 |

**Table S5.** **Number of SNP effects by region in the marine medaka genome.**

| Region | Number | Percentage (%) |
| --- | --- | --- |
| DOWNSTREAM | 645,016 | 12.677 |
| EXON | 100,412 | 1.973 |
| INTERGENIC | 1,866,032 | 36.673 |
| INTRON | 1,756,451 | 34.52 |
| SPLICE_SITE_ACCEPTOR | 110 | 0.002 |
| SPLICE_SITE_DONOR | 141 | 0.003 |
| SPLICE_SITE_REGION | 13,401 | 0.263 |
| UPSTREAM | 647,922 | 12.734 |
| UTR_3_PRIME | 45,121 | 0.887 |
| UTR_5_PRIME | 13,624 | 0.268 |

**Table S6. Genome completeness as measured by CEGMA and BUSCO.**

|  | completeness |
| --- | --- |
| Complete CEGMA | 93.15% |
| Complete + partial CEGMA | 96.37% |
| Complete and single-copy BUSCOs | 94.90% |
| Complete and duplicated BUSCOs | 7% |
| Fragmented BUSCOs | 0.80% |
| Missing BUSCOs | 4.30% |

**Table S7. Statistical of predicted functional genes in public protein databases.**

| Batabase | Annotated Number | Annotated Percent(%) |
| --- | --- | --- |
| NR | 23,344 | 90.8 |
| Swiss-Prot | 21,788 | 84.8 |
| KEGG | 20,136 | 78.4 |
| InterPro | 25,424 | 98.9 |
| Pfam | 19,739 | 76.8 |
| GO | 23,572 | 91.7 |
| Annotated | 25,486 | 99.2 |
| Total | 25,699 | - |

**Table S8. The number of all kinds of non-coding RNA.**

| Type | | Copy(w*) | Average length(bp) | Total length(bp) | % of genome |
| --- | --- | --- | --- | --- | --- |
| miRNA | | 926 | 98.60 | 91,300 | 0.010815 |
| tRNA | | 1,916 | 74.58 | 142,886 | 0.016926 |
| rRNA | rRNA | 825 | 94.02 | 77,567 | 0.009189 |
|  | 18S | 421 | 98.21 | 41,348 | 0.004898 |
|  | 28S | 320 | 87.18 | 27,899 | 0.003305 |
|  | 5.8S | 2 | 113.50 | 227 | 0.000027 |
|  | 5S | 82 | 98.70 | 8,093 | 0.000959 |
| snRNA | snRNA | 295 | 124.32 | 36,674 | 0.004344 |
|  | CD-box | 105 | 97.10 | 10,196 | 0.001208 |
|  | HACA-box | 73 | 141.53 | 10,332 | 0.001224 |
|  | splicing | 107 | 134.19 | 14,358 | 0.001208 |
| lncRNA |  | 2,474 | 1,453.51 | 3,595,986 | 0.425981 |

Note: w*, whole genome annotation

**Table S9. Summary statistics of repeat elements*.***

| **Repetitive element** | **Previous assembly** | | **New assembly** | | ***Oryzias latipes*** | |
| --- | --- | --- | --- | --- | --- | --- |
| Order | Total bp | % in genome | Total bp | % in genome | Total bp | % in genome |
| DNA | 87,942,605 | 11.28 | 97,185,984 | 11.51 | 108,105,878 | 14.73 |
| LINE | 87,774,678 | 11.26 | 132,643,381 | 15.71 | 111,784,681 | 15.23 |
| SINE | 22,062,094 | 2.83 | 16,501,602 | 1.95 | 3,789,541 | 0.52 |
| LTR | 65,190,808 | 8.36 | 88,672,011 | 10.5 | 70,723,500 | 9.63 |
| Simple_repeat | 4,496,670 | 0.58 | 2,978,379 | 0.35 | 5,153,572 | 0.7 |
| Unknown | 50,190,505 | 6.44 | 37,890,867 | 4.49 | 17,796,822 | 2.42 |
| Total | 262,476,399 | 33.67 | 326,618,780 | 38.69 | 277,771,502 | 37.84 |

**Table S10. Significantly over-represented Gene Ontology (GO) terms among *O. melastigma*-specific genes compared with *Oryzias latipes, Nothobranchius furzeri* and *Xiphophorus maculatus*.** “X” is the number of *O. melastigma*-specific genes assigned to that GO term. The GO terms with corrected *P-*value bellow 0.05 are selected as significantly enriched groups.

| **GO ID** | **Term** | **Class** | **X** | ***P-*value** | **Adj.**  ***P-*value** |
| --- | --- | --- | --- | --- | --- |
| GO:0006915 | apoptotic process | BP | 14 | 0.000 | 8.82E-06 |
| GO:0012501 | programmed cell death | BP | 14 | 0.000 | 8.82E-06 |
| GO:0008219 | cell death | BP | 14 | 0.000 | 8.82E-06 |
| GO:0016265 | death | BP | 14 | 0.000 | 8.82E-06 |
| GO:0042981 | regulation of apoptotic process | BP | 12 | 0.000 | 1.36E-05 |
| GO:0010941 | regulation of cell death | BP | 12 | 0.000 | 1.36E-05 |
| GO:0043067 | regulation of programmed cell death | BP | 12 | 0.000 | 1.36E-05 |
| GO:0043065 | positive regulation of apoptotic process | BP | 4 | 0.000 | 0.000775 |
| GO:0010942 | positive regulation of cell death | BP | 4 | 0.000 | 0.000775 |
| GO:0043068 | positive regulation of programmed cell death | BP | 4 | 0.000 | 0.000775 |
| GO:0051085 | chaperone mediated protein folding requiring cofactor | BP | 3 | 0.000 | 0.001623 |
| GO:0006458 | 'de novo' protein folding | BP | 3 | 0.000 | 0.001623 |
| GO:0051084 | 'de novo' posttranslational protein folding | BP | 3 | 0.000 | 0.001623 |
| GO:0061077 | chaperone-mediated protein folding | BP | 3 | 0.000 | 0.001623 |
| GO:0003896 | DNA primase activity | MF | 2 | 0.001 | 0.039107 |
| GO:0006269 | DNA replication, synthesis of RNA primer | BP | 2 | 0.001 | 0.039107 |

**Table S11. The list of 44 expanded gene families and 46 contracted gene families that appeared unique to *Oryzias melastigma*.**

| **Type** | **Gene locus tag** |
| --- | --- |
| expanded genes | FQA47_025340, FQA47_025325, FQA47_025332, FQA47_011478, FQA47_000932, FQA47_000927, FQA47_007902, FQA47_022832, FQA47_004502, FQA47_004580, FQA47_002591, FQA47_002593, FQA47_010953, FQA47_010917, FQA47_009019, FQA47_009179, FQA47_003645, FQA47_022363, FQA47_022368, FQA47_022349, FQA47_020077, FQA47_020059, FQA47_018881, FQA47_018879, FQA47_005310, FQA47_016124, FQA47_020502, FQA47_020499, FQA47_020800, FQA47_008116, FQA47_017169, FQA47_007521, FQA47_016195, FQA47_017781, FQA47_003366, FQA47_003352, FQA47_023260, FQA47_023261, FQA47_014020, FQA47_019799, FQA47_022406, FQA47_025235, FQA47_025192, FQA47_012320, FQA47_017726, FQA47_015970, FQA47_010433, FQA47_018335, FQA47_015082, FQA47_022318, FQA47_007414, FQA47_019229, FQA47_019232, FQA47_018650, FQA47_021782, FQA47_021252, FQA47_014022, FQA47_001751, FQA47_005863, FQA47_018533, FQA47_018520, FQA47_010714, FQA47_010720, FQA47_020022, FQA47_008426, FQA47_005023, FQA47_005555, FQA47_022654, FQA47_006355, FQA47_000690, FQA47_019488, FQA47_021160, FQA47_013073, FQA47_013083, FQA47_008594, FQA47_024445, FQA47_010139, FQA47_010129, FQA47_000919, FQA47_000912, FQA47_016220, FQA47_016216, FQA47_016228, FQA47_016214, FQA47_016232, FQA47_016227, FQA47_006282, FQA47_008680, FQA47_002252, FQA47_024349, FQA47_016495, FQA47_007512, FQA47_007480, FQA47_007492, FQA47_024823, FQA47_015622, FQA47_015612, FQA47_015535, FQA47_002034, FQA47_001963, FQA47_001979, FQA47_002094, FQA47_009643, FQA47_009801, FQA47_020625, FQA47_020678, FQA47_017311, FQA47_021552, FQA47_001837, FQA47_001905, FQA47_020738, FQA47_023288, FQA47_013970, FQA47_016965, FQA47_016923, FQA47_016968, FQA47_019338, FQA47_003240, FQA47_004451, FQA47_025093, FQA47_025096, FQA47_017378, FQA47_017978, FQA47_019703, FQA47_019680, FQA47_002085, FQA47_009721, FQA47_009734, FQA47_025749, FQA47_025753, FQA47_025527, FQA47_018862, FQA47_018861, FQA47_013772, FQA47_019426, FQA47_019431, FQA47_012229, FQA47_025037, FQA47_025038, FQA47_003243, FQA47_015366, FQA47_015351, FQA47_021677, FQA47_021683, FQA47_016040, FQA47_009164, FQA47_010631, FQA47_010230, FQA47_001501, FQA47_001442, FQA47_008357, FQA47_008393, FQA47_004237, FQA47_004198, FQA47_013591, FQA47_018773, FQA47_018790, FQA47_018789, FQA47_017363, FQA47_022697, FQA47_022682, FQA47_022731, FQA47_023397, FQA47_000485, FQA47_000370, FQA47_007753, FQA47_007705, FQA47_007750, FQA47_024287, FQA47_019093, FQA47_019092, FQA47_013716, FQA47_019677, FQA47_019671, FQA47_009368, FQA47_009353, FQA47_016824, FQA47_004447, FQA47_004433, FQA47_004435, FQA47_006179, FQA47_000757, FQA47_005940, FQA47_005112, FQA47_011649, FQA47_013987, FQA47_005063, FQA47_012285, FQA47_005184, FQA47_013910, FQA47_006764, FQA47_006249, FQA47_014981, FQA47_016392, FQA47_016716, FQA47_025368, FQA47_004639, FQA47_007223, FQA47_018976, FQA47_011142, FQA47_019713, FQA47_007430, FQA47_021235, FQA47_021217, FQA47_009636, FQA47_009657, FQA47_003355, FQA47_008779, FQA47_006934, FQA47_016943, FQA47_002512, FQA47_003226, FQA47_003245, FQA47_015730, FQA47_007506, FQA47_013800, FQA47_012880, FQA47_012853, FQA47_001938, FQA47_023574, FQA47_016588, FQA47_007878, FQA47_009120, FQA47_009121, FQA47_010571, FQA47_010547, FQA47_019525, FQA47_003310, FQA47_003309, FQA47_020695, FQA47_020768, FQA47_020728, FQA47_020741, FQA47_025003, FQA47_009690, FQA47_009742, FQA47_009672, FQA47_009769, FQA47_000716, FQA47_000733, FQA47_009990, FQA47_009963, FQA47_009981, FQA47_020356, FQA47_020377, FQA47_020362, FQA47_015027, FQA47_015045, FQA47_023128, FQA47_023125, FQA47_020389, FQA47_020418, FQA47_020399, FQA47_020443, FQA47_023795, FQA47_023801, FQA47_004142, FQA47_004145, FQA47_014521, FQA47_024925, FQA47_008321, FQA47_008284, FQA47_014257, FQA47_014252, FQA47_008155, FQA47_016952, FQA47_011536, FQA47_012788, FQA47_021594, FQA47_021596, FQA47_007277, FQA47_007281, FQA47_007272, FQA47_007282, FQA47_022125, FQA47_022129, FQA47_022095, FQA47_022114, FQA47_022115, FQA47_022127, FQA47_022117, FQA47_022105, FQA47_022097, FQA47_022116, FQA47_022091, FQA47_021195, FQA47_021729, FQA47_021746, FQA47_005393, FQA47_020770, FQA47_006823, FQA47_009242, FQA47_009189, FQA47_009170, FQA47_000177, FQA47_012491, FQA47_001602, FQA47_023761, FQA47_023760, FQA47_003502, FQA47_003527, FQA47_003602, FQA47_003581, FQA47_003518, FQA47_015048, FQA47_001231, FQA47_001233, FQA47_003628, FQA47_003610, FQA47_003618, FQA47_003620, FQA47_009419, FQA47_009497, FQA47_016967, FQA47_003333, FQA47_007720, FQA47_017270, FQA47_012457, FQA47_007296, FQA47_007254, FQA47_018059, FQA47_014657, FQA47_015605, FQA47_023755, FQA47_023758, FQA47_012987, FQA47_020337, FQA47_021103, FQA47_021111, FQA47_023161, FQA47_013051, FQA47_005734, FQA47_023933, FQA47_005424, FQA47_017714, FQA47_019828, FQA47_006739, FQA47_001262, FQA47_023411, FQA47_023403, FQA47_022998, FQA47_014233, FQA47_013684, FQA47_000619, FQA47_004465, FQA47_004464, FQA47_001913, FQA47_008416, FQA47_008429, FQA47_002807, FQA47_023631, FQA47_008238, FQA47_004144, FQA47_004140, FQA47_004138 |
| contracted genes | FQA47_018121, FQA47_011294, FQA47_004944, FQA47_000740, FQA47_001575, FQA47_021724, FQA47_021739, FQA47_016033, FQA47_017535, FQA47_019745, FQA47_021269, FQA47_021060, FQA47_017855, FQA47_007436, FQA47_001893, FQA47_017346, FQA47_011492, FQA47_023057, FQA47_019920, FQA47_002632, FQA47_015133, FQA47_003891, FQA47_014319, FQA47_010289, FQA47_021475, FQA47_025001, FQA47_002116, FQA47_023074, FQA47_023091, FQA47_000908, FQA47_015834, FQA47_022044, FQA47_015391, FQA47_001534, FQA47_023081, FQA47_023064, FQA47_005341, FQA47_013430, FQA47_007685, FQA47_012299, FQA47_007463 |

**Table S12. KEGG pathway results of expanded gene families.**

| Map_ID | Title | # in expanded gene families | # in the genome | Adj. *P-*value |
| --- | --- | --- | --- | --- |
| map04020 | Calcium signaling pathway | 33 | 470 | 3.48E-18 |
| map02010 | ABC transporters | 18 | 66 | 5.78E-16 |
| map04514 | Cell adhesion molecules (CAMs) | 24 | 345 | 50E-13 |
| map04713 | Circadian entrainment | 26 | 251 | 18E-12 |
| map04024 | cAMP signaling pathway | 27 | 458 | 1.94E-11 |
| map04720 | Long-term potentiation | 18 | 162 | 2.49E-09 |
| map05322 | Systemic lupus erythematosus | 14 | 118 | 10E-07 |
| map05030 | Cocaine addiction | 12 | 100 | 9.23E-07 |
| map05033 | Nicotine addiction | 12 | 100 | 9.23E-07 |
| map05014 | Amyotrophic lateral sclerosis (ALS) | 12 | 106 | 1.60E-06 |
| map05010 | Alzheimer's disease | 19 | 293 | 3.09E-06 |
| map05414 | Dilated cardiomyopathy | 16 | 229 | 9.50E-06 |
| map05162 | Measles | 15 | 216 | 2.08E-05 |
| map05031 | Amphetamine addiction | 12 | 150 | 4.78E-05 |
| map05310 | Asthma | 7 | 45 | 5.37E-05 |
| map05150 | Staphylococcus aureus infection | 10 | 107 | 6.44E-05 |
| map04360 | Axon guidance | 18 | 427 | 0.00027466 |
| map04672 | Intestinal immune network for IgA production | 7 | 69 | 0.00076427 |
| map05410 | Hypertrophic cardiomyopathy (HCM) | 12 | 205 | 0.00077437 |
| map05416 | Viral myocarditis | 10 | 152 | 0.00104181 |
| map05330 | Allograft rejection | 7 | 80 | 0.00165838 |
| map05034 | Alcoholism | 12 | 227 | 0.00171815 |
| map05320 | Autoimmune thyroid disease | 7 | 92 | 0.00354251 |
| map04931 | Insulin resistance | 11 | 228 | 0.00585567 |
| map04724 | Glutamatergic synapse | 12 | 266 | 0.0060967 |
| map00310 | Lysine degradation | 7 | 118 | 0.01322557 |
| map04391 | Hippo signaling pathway - fly | 8 | 155 | 0.01561449 |
| map04340 | Hedgehog signaling pathway | 6 | 94 | 0.01674624 |
| map05144 | Malaria | 6 | 102 | 0.02402918 |
| map05323 | Rheumatoid arthritis | 7 | 138 | 0.02684838 |
| map04520 | Adherens junction | 9 | 220 | 0.03524112 |
| map04150 | mTOR signaling pathway | 6 | 114 | 0.03675895 |
| map04976 | Bile secretion | 7 | 152 | 0.04030428 |
| map04921 | Oxytocin signaling pathway | 12 | 350 | 0.04240963 |
| map04080 | Neuroactive ligand-receptor interaction | 18 | 616 | 0.0470583 |
| map04914 | Progesterone-mediated oocyte maturation | 7 | 161 | 0.04934049 |

**Table S13. GO functional enrichment results for expanded gene families.**

| GO_ID | GO_term | Class | # in expanded gene families | # in the genome | Adj. *P-*value |
| --- | --- | --- | --- | --- | --- |
| GO:0042623 | ATPase activity, coupled | MF | 44 | 576 | 9.51E-33 |
| GO:0016887 | ATPase activity | MF | 45 | 616 | 15E-31 |
| GO:0055085 | transmembrane transport | BP | 58 | 1084 | 2.27E-25 |
| GO:0043492 | ATPase activity, coupled to movement of substances | MF | 35 | 225 | 1.74E-23 |
| GO:0004012 | phospholipid-translocating ATPase activity | MF | 17 | 27 | 3.38E-23 |
| GO:0015914 | phospholipid transport | BP | 17 | 27 | 3.38E-23 |
| GO:0005509 | calcium ion binding | MF | 42 | 938 | 3.99E-13 |
| GO:0017111 | nucleoside-triphosphatase activity | MF | 53 | 1370 | 1.40E-12 |
| GO:0005215 | transporter activity | MF | 106 | 3828 | 4.32E-12 |
| GO:0005219 | ryanodine-sensitive calcium-release channel activity | MF | 7 | 7 | 7.25E-12 |
| GO:0000287 | magnesium ion binding | MF | 17 | 108 | 1.54E-11 |
| GO:0032559 | adenyl ribonucleotide binding | MF | 74 | 2342 | 1.63E-11 |
| GO:0005524 | ATP binding | MF | 73 | 2337 | 4.92E-11 |
| GO:0097367 | carbohydrate derivative binding | MF | 89 | 3111 | 6.52E-11 |
| GO:0032555 | purine ribonucleotide binding | MF | 87 | 3017 | 6.90E-11 |
| GO:0035639 | purine ribonucleoside triphosphate binding | MF | 86 | 2999 | 1.28E-10 |
| GO:0032550 | purine ribonucleoside binding | MF | 86 | 3000 | 1.28E-10 |
| GO:0042626 | ATPase activity, coupled to transmembrane movement of substances | MF | 20 | 201 | 4.77E-10 |
| GO:0007156 | homophilic cell adhesion | BP | 23 | 280 | 6.99E-10 |
| GO:0000166 | nucleotide binding | MF | 89 | 3250 | 9.41E-10 |
| GO:0036094 | small molecule binding | MF | 91 | 3353 | 9.46E-10 |
| GO:0004970 | ionotropic glutamate receptor activity | MF | 10 | 39 | 3.88E-09 |
| GO:0005515 | protein binding | MF | 155 | 7046 | 4.79E-09 |
| GO:0005234 | extracellular-glutamate-gated ion channel activity | MF | 10 | 43 | 1.06E-08 |
| GO:0015267 | channel activity | MF | 61 | 2005 | 1.20E-08 |
| GO:0005216 | ion channel activity | MF | 60 | 1964 | 1.34E-08 |
| GO:0016020 | membrane | CC | 213 | 10871 | 1.38E-08 |
| GO:0007416 | synapse assembly | BP | 6 | 9 | 1.55E-08 |
| GO:0005262 | calcium channel activity | MF | 19 | 349 | 26E-08 |
| GO:0022857 | transmembrane transporter activity | MF | 86 | 3296 | 3.69E-08 |
| GO:0005248 | voltage-gated sodium channel activity | MF | 9 | 39 | 6.91E-08 |
| GO:0044765 | single-organism transport | BP | 102 | 4222 | 1.07E-07 |
| GO:0001518 | voltage-gated sodium channel complex | CC | 9 | 44 | 2.03E-07 |
| GO:0022892 | substrate-specific transporter activity | MF | 85 | 3348 | 2.08E-07 |
| GO:0006810 | transport | BP | 114 | 5066 | 9.82E-07 |
| GO:0051179 | localization | BP | 118 | 5313 | 14E-06 |
| GO:0043168 | anion binding | MF | 88 | 3638 | 1.54E-06 |
| GO:0005488 | binding | MF | 248 | 13922 | 1.98E-06 |
| GO:0006811 | ion transport | BP | 77 | 3112 | 4.37E-06 |
| GO:0007155 | cell adhesion | BP | 34 | 1051 | 1.62E-05 |
| GO:0004725 | protein tyrosine phosphatase activity | MF | 11 | 120 | 2.01E-05 |
| GO:0015276 | ligand-gated ion channel activity | MF | 18 | 414 | 28E-05 |
| GO:0043167 | ion binding | MF | 136 | 6776 | 5.62E-05 |
| GO:0006874 | cellular calcium ion homeostasis | BP | 7 | 50 | 1.00E-04 |
| GO:0016787 | hydrolase activity | MF | 78 | 3465 | 0.00023703 |
| GO:0006875 | cellular metal ion homeostasis | BP | 9 | 103 | 0.00023703 |
| GO:0022836 | gated channel activity | MF | 41 | 1518 | 0.00038846 |
| GO:0070588 | calcium ion transmembrane transport | BP | 15 | 318 | 0.00075143 |
| GO:0015075 | ion transmembrane transporter activity | MF | 69 | 3087 | 0.00099687 |
| GO:0022891 | substrate-specific transmembrane transporter activity | MF | 70 | 3169 | 0.00127724 |
| GO:0006470 | protein dephosphorylation | BP | 11 | 194 | 0.00130826 |
| GO:0007218 | neuropeptide signaling pathway | BP | 10 | 169 | 0.00177613 |
| GO:0090129 | positive regulation of synapse maturation | BP | 2 | 2 | 0.00196278 |
| GO:0016791 | phosphatase activity | MF | 14 | 316 | 0.00198643 |
| GO:0006820 | anion transport | BP | 20 | 611 | 0.00205539 |
| GO:0042578 | phosphoric ester hydrolase activity | MF | 15 | 425 | 0.00463548 |
| GO:0046872 | metal ion binding | MF | 76 | 3688 | 0.00549468 |
| GO:0022804 | active transmembrane transporter activity | MF | 26 | 938 | 0.00575742 |
| GO:0007269 | neurotransmitter secretion | BP | 3 | 14 | 0.00713615 |
| GO:0051965 | positive regulation of synapse assembly | BP | 2 | 4 | 0.00870097 |
| GO:0016301 | kinase activity | MF | 32 | 1301 | 0.01474408 |
| GO:0005261 | cation channel activity | MF | 36 | 1515 | 0.01488094 |
| GO:0004674 | protein serine/threonine kinase activity | MF | 11 | 287 | 0.02102375 |
| GO:0009986 | cell surface | CC | 3 | 21 | 0.02194043 |
| GO:0016774 | phosphotransferase activity, carboxyl group as acceptor | MF | 6 | 106 | 0.02878443 |
| GO:0004713 | protein tyrosine kinase activity | MF | 18 | 634 | 0.02986061 |
| GO:0016021 | integral component of membrane | CC | 126 | 7084 | 0.0298659 |
| GO:0006464 | cellular protein modification process | BP | 32 | 1367 | 0.03525349 |
| GO:0004896 | cytokine receptor activity | MF | 10 | 270 | 0.03817669 |
| GO:0030246 | carbohydrate binding | MF | 8 | 189 | 0.03957169 |
| GO:0006814 | sodium ion transport | BP | 11 | 323 | 0.04617457 |
| GO:0033036 | macromolecule localization | BP | 20 | 760 | 0.04840491 |

**Table S14. Positively selected genes in the *O. melastigma*.**

| ***O. melastigma* Gene locus tag** | **Sites** | ***P-*value** | **Adj. *P-*value** |
| --- | --- | --- | --- |
| FQA47_015479 | 3 | 9.3E-05 | 9.9E-04 |
| FQA47_012111 | 1 | 6.8E-03 | 3.5E-02 |
| FQA47_024405 | 1 | 2.0E-06 | 3.4E-05 |
| FQA47_000976 | 1 | 1.3E-05 | 1.8E-04 |
| FQA47_020210 | 1 | 9.8E-06 | 1.4E-04 |
| FQA47_018475 | 1 | 6.8E-03 | 3.5E-02 |
| FQA47_020987 | 1 | 1E-05 | 1.6E-04 |
| FQA47_015896 | 1 | 6.3E-03 | 3.3E-02 |
| FQA47_024034 | 1 | 1E-03 | 8.0E-03 |
| FQA47_001295 | 1 | 4.3E-04 | 3.7E-03 |
| FQA47_015831 | 1 | 1.0E-11 | 8.4E-10 |
| FQA47_018400 | 1 | 1.5E-07 | 3.7E-06 |
| FQA47_016915 | 1 | 6.5E-05 | 7.2E-04 |
| FQA47_007583 | 2 | 4.7E-06 | 7.4E-05 |
| FQA47_004965 | 1 | 1.2E-05 | 1.8E-04 |
| FQA47_023472 | 1 | 2.7E-05 | 3.4E-04 |
| FQA47_022945 | 3 | 9.6E-07 | 1.8E-05 |
| FQA47_011281 | 1 | 3.9E-05 | 4.6E-04 |
| FQA47_021230 | 1 | 7E-09 | 2.6E-07 |
| FQA47_012300 | 1 | 3.4E-07 | 7.2E-06 |
| FQA47_016316 | 1 | 9E-03 | 4.5E-02 |
| FQA47_008291 | 3 | 6.2E-06 | 9.5E-05 |
| FQA47_000015 | 1 | 1.9E-06 | 3.3E-05 |
| FQA47_025532 | 1 | 5E-07 | 1.0E-05 |
| FQA47_018653 | 1 | 7.6E-07 | 1.5E-05 |
| FQA47_016059 | 1 | 8.3E-04 | 6.3E-03 |
| FQA47_001654 | 1 | 2E-09 | 8.3E-08 |
| FQA47_008998 | 1 | 1.3E-05 | 1.8E-04 |
| FQA47_024487 | 5 | 3E-06 | 5.2E-05 |
| FQA47_022653 | 1 | 2.9E-05 | 3.6E-04 |
| FQA47_012052 | 1 | 0.0E+00 | 0.0E+00 |
| FQA47_024332 | 1 | 4.8E-04 | 4.0E-03 |
| FQA47_017682 | 1 | 3.9E-04 | 3.4E-03 |
| FQA47_019106 | 1 | 2E-03 | 1.4E-02 |
| FQA47_021920 | 1 | 2.2E-12 | 2.2E-10 |
| FQA47_012753 | 1 | 1.6E-09 | 6.5E-08 |
| FQA47_022520 | 1 | 7.4E-04 | 5.8E-03 |
| FQA47_007793 | 1 | 9E-03 | 4.4E-02 |
| FQA47_020507 | 1 | 1.0E-03 | 7.3E-03 |
| FQA47_003515 | 1 | 0.0E+00 | 0.0E+00 |
| FQA47_005515 | 1 | 8.2E-05 | 8.9E-04 |
| FQA47_013379 | 1 | 2.0E-06 | 3.4E-05 |
| FQA47_018100 | 1 | 8.6E-10 | 3.8E-08 |
| FQA47_006021 | 2 | 3.2E-03 | 1.9E-02 |
| FQA47_005283 | 1 | 2E-10 | 1E-08 |
| FQA47_002009 | 1 | 8.6E-06 | 1.3E-04 |
| FQA47_000363 | 1 | 1E-04 | 1E-03 |
| FQA47_016041 | 1 | 2.3E-04 | 2.2E-03 |
| FQA47_013713 | 1 | 9E-05 | 9.8E-04 |
| FQA47_018213 | 1 | 1.8E-08 | 5.8E-07 |
| FQA47_003225 | 2 | 1.8E-05 | 2.4E-04 |
| FQA47_014804 | 1 | 8.7E-11 | 5.5E-09 |
| FQA47_023470 | 1 | 2.8E-05 | 3.5E-04 |
| FQA47_016068 | 1 | 8.7E-03 | 4.3E-02 |
| FQA47_013680 | 1 | 1.8E-05 | 2.4E-04 |
| FQA47_007017 | 1 | 1.4E-04 | 1.4E-03 |
| FQA47_020034 | 2 | 4.7E-08 | 1.3E-06 |
| FQA47_020637 | 1 | 4.2E-08 | 1.2E-06 |
| FQA47_008451 | 1 | 1.6E-04 | 1.6E-03 |
| FQA47_001618 | 1 | 8.9E-16 | 3.7E-13 |
| FQA47_022626 | 1 | 1.3E-05 | 1.8E-04 |
| FQA47_025086 | 1 | 4.7E-03 | 2.6E-02 |
| FQA47_022183 | 2 | 2.8E-11 | 2.0E-09 |
| FQA47_008505 | 2 | 9E-04 | 6.7E-03 |
| FQA47_013340 | 1 | 4.0E-05 | 4.7E-04 |
| FQA47_015396 | 3 | 9.0E-04 | 6.7E-03 |
| FQA47_014474 | 2 | 2.3E-03 | 1.5E-02 |
| FQA47_009330 | 1 | 4.9E-05 | 5.6E-04 |
| FQA47_025185 | 3 | 1.0E-02 | 5.0E-02 |
| FQA47_015046 | 1 | 7.4E-03 | 3.8E-02 |
| FQA47_022519 | 1 | 9.8E-04 | 7.2E-03 |
| FQA47_006917 | 1 | 1.4E-04 | 1.4E-03 |
| FQA47_013043 | 1 | 1.0E-02 | 4.8E-02 |
| FQA47_005652 | 1 | 1.2E-03 | 8.6E-03 |
| FQA47_000286 | 1 | 8.8E-05 | 9.5E-04 |
| FQA47_010822 | 2 | 3.7E-03 | 2.2E-02 |
| FQA47_011483 | 1 | 8.6E-09 | 3.0E-07 |
| FQA47_025472 | 2 | 3.2E-09 | 1.2E-07 |
| FQA47_002992 | 1 | 3.4E-06 | 5.5E-05 |
| FQA47_019118 | 1 | 4.6E-08 | 1.3E-06 |
| FQA47_006949 | 1 | 6.5E-06 | 9.9E-05 |
| FQA47_008941 | 1 | 7.2E-03 | 3.7E-02 |
| FQA47_014059 | 2 | 5E-03 | 2.8E-02 |
| FQA47_003267 | 1 | 2.8E-07 | 6.2E-06 |
| FQA47_004893 | 1 | 1.0E-08 | 3.5E-07 |
| FQA47_003520 | 1 | 2.2E-04 | 2.0E-03 |
| FQA47_001137 | 3 | 2.9E-03 | 1.8E-02 |
| FQA47_013341 | 1 | 8.6E-04 | 6.5E-03 |
| FQA47_006833 | 1 | 4E-04 | 3.5E-03 |
| FQA47_015489 | 1 | 4.4E-16 | 1.9E-13 |
| FQA47_000962 | 1 | 3.6E-05 | 4.3E-04 |
| FQA47_004384 | 2 | 1.7E-03 | 1E-02 |
| FQA47_006191 | 1 | 1.2E-03 | 8.5E-03 |
| FQA47_000455 | 1 | 3.2E-06 | 5.3E-05 |
| FQA47_015151 | 1 | 7.3E-03 | 3.7E-02 |
| FQA47_016746 | 1 | 1.8E-08 | 5.7E-07 |
| FQA47_022890 | 1 | 2.8E-05 | 3.5E-04 |
| FQA47_018937 | 1 | 2.3E-10 | 1.2E-08 |
| FQA47_012259 | 1 | 3.9E-05 | 4.7E-04 |
| FQA47_021817 | 1 | 5.2E-04 | 4.3E-03 |
| FQA47_011754 | 1 | 1.2E-07 | 3.0E-06 |
| FQA47_020776 | 1 | 6.2E-12 | 5.4E-10 |
| FQA47_006941 | 1 | 6E-11 | 4.0E-09 |
| FQA47_000178 | 1 | 4E-04 | 3.5E-03 |
| FQA47_012217 | 1 | 2.2E-03 | 1.4E-02 |
| FQA47_001266 | 1 | 8.0E-03 | 4.0E-02 |
| FQA47_022987 | 1 | 2.4E-04 | 2.2E-03 |
| FQA47_007077 | 1 | 3.0E-04 | 2.7E-03 |
| FQA47_000967 | 1 | 1.9E-04 | 1.8E-03 |
| FQA47_000963 | 3 | 6.3E-04 | 5.0E-03 |
| FQA47_001594 | 1 | 7.5E-07 | 1.5E-05 |
| FQA47_013153 | 1 | 1.9E-06 | 3.4E-05 |
| FQA47_012813 | 1 | 7.4E-03 | 3.8E-02 |
| FQA47_006338 | 1 | 2.6E-07 | 5.6E-06 |
| FQA47_023891 | 1 | 6E-15 | 1.5E-12 |
| FQA47_006654 | 1 | 6.2E-11 | 4.0E-09 |
| FQA47_002979 | 2 | 1.7E-07 | 4.0E-06 |
| FQA47_012553 | 1 | 5.8E-05 | 6.5E-04 |
| FQA47_010970 | 1 | 8.7E-04 | 6.5E-03 |
| FQA47_014193 | 2 | 9E-08 | 2.3E-06 |
| FQA47_016996 | 1 | 6.8E-04 | 5.4E-03 |
| FQA47_016947 | 1 | 3.0E-03 | 1.8E-02 |
| FQA47_016646 | 1 | 1.2E-14 | 2.7E-12 |
| FQA47_023815 | 1 | 1.9E-04 | 1.8E-03 |
| FQA47_013578 | 1 | 8.2E-05 | 8.9E-04 |
| FQA47_002545 | 1 | 8.4E-04 | 6.4E-03 |
| FQA47_002594 | 3 | 3.3E-10 | 1.6E-08 |
| FQA47_014093 | 1 | 1.9E-07 | 4.3E-06 |
| FQA47_008026 | 1 | 5E-11 | 3.5E-09 |
| FQA47_016484 | 1 | 1E-03 | 7.7E-03 |
| FQA47_012756 | 1 | 1.2E-03 | 8.5E-03 |
| FQA47_014828 | 2 | 3.2E-03 | 1.9E-02 |
| FQA47_010675 | 1 | 3.2E-03 | 1.9E-02 |
| FQA47_018565 | 1 | 5.3E-07 | 1E-05 |
| FQA47_016177 | 1 | 1.8E-10 | 9.8E-09 |
| FQA47_012795 | 2 | 1.2E-03 | 8.5E-03 |
| FQA47_018866 | 1 | 2.9E-03 | 1.8E-02 |
| FQA47_018835 | 3 | 3.7E-14 | 6.8E-12 |
| FQA47_021073 | 1 | 1.9E-12 | 1.9E-10 |
| FQA47_003416 | 2 | 2.6E-03 | 1.6E-02 |
| FQA47_024883 | 1 | 3.0E-04 | 2.8E-03 |
| FQA47_025458 | 1 | 6.6E-04 | 5.2E-03 |
| FQA47_008075 | 1 | 7E-04 | 5.6E-03 |
| FQA47_007031 | 1 | 1.5E-03 | 1.0E-02 |
| FQA47_002620 | 1 | 4.7E-09 | 1.7E-07 |
| FQA47_019362 | 1 | 8.5E-04 | 6.4E-03 |
| FQA47_017484 | 1 | 1.0E-07 | 2.6E-06 |
| FQA47_024834 | 3 | 5.4E-06 | 8.4E-05 |
| FQA47_000916 | 1 | 1.7E-04 | 1.7E-03 |
| FQA47_007246 | 1 | 2E-03 | 1.4E-02 |
| FQA47_003410 | 1 | 4E-04 | 3.5E-03 |
| FQA47_017734 | 1 | 7.2E-12 | 6.0E-10 |
| FQA47_014918 | 1 | 2.2E-15 | 6.8E-13 |
| FQA47_022263 | 1 | 6.7E-11 | 4.2E-09 |
| FQA47_009337 | 1 | 9.0E-03 | 4.4E-02 |
| FQA47_008323 | 2 | 1.6E-10 | 9E-09 |
| FQA47_006534 | 1 | 8.6E-04 | 6.4E-03 |
| FQA47_008513 | 1 | 1E-05 | 1.5E-04 |
| FQA47_008364 | 1 | 8.0E-05 | 8.7E-04 |
| FQA47_020151 | 1 | 1.3E-10 | 7.5E-09 |
| FQA47_005935 | 1 | 3.3E-16 | 1.7E-13 |
| FQA47_011672 | 1 | 7E-07 | 1.4E-05 |
| FQA47_012042 | 1 | 7E-07 | 1.4E-05 |
| FQA47_008268 | 1 | 1.8E-04 | 1.8E-03 |
| FQA47_022249 | 1 | 2.5E-03 | 1.6E-02 |
| FQA47_016199 | 1 | 8.3E-04 | 6.3E-03 |
| FQA47_014438 | 1 | 8.8E-07 | 1.7E-05 |
| FQA47_002466 | 1 | 3.4E-03 | 2.0E-02 |
| FQA47_008100 | 2 | 1.5E-08 | 4.9E-07 |
| FQA47_004948 | 1 | 2E-14 | 4E-12 |
| FQA47_016283 | 1 | 3.7E-08 | 1E-06 |
| FQA47_017437 | 1 | 1.5E-04 | 1.5E-03 |
| FQA47_003732 | 2 | 2.3E-03 | 1.5E-02 |
| FQA47_011146 | 2 | 7.9E-10 | 3.6E-08 |
| FQA47_017867 | 1 | 1.7E-05 | 2.3E-04 |
| FQA47_003547 | 1 | 6.6E-03 | 3.5E-02 |
| FQA47_009212 | 1 | 1.7E-04 | 1.7E-03 |
| FQA47_015331 | 2 | 3E-05 | 3.8E-04 |
| FQA47_010972 | 3 | 1E-14 | 2.6E-12 |
| FQA47_011120 | 1 | 2.5E-03 | 1.6E-02 |
| FQA47_022757 | 3 | 9.4E-07 | 1.8E-05 |
| FQA47_010941 | 1 | 4.2E-04 | 3.6E-03 |
| FQA47_012462 | 1 | 6.2E-05 | 6.9E-04 |
| FQA47_005868 | 4 | 1.2E-09 | 5.0E-08 |
| FQA47_012793 | 1 | 8.9E-08 | 2.3E-06 |
| FQA47_012018 | 2 | 8.3E-06 | 1.2E-04 |
| FQA47_013410 | 1 | 1.3E-08 | 4E-07 |
| FQA47_003107 | 1 | 5.2E-04 | 4.2E-03 |
| FQA47_000118 | 1 | 4.7E-12 | 4E-10 |
| FQA47_021917 | 1 | 8E-05 | 8.8E-04 |
| FQA47_016504 | 1 | 0.0E+00 | 0.0E+00 |
| FQA47_012325 | 1 | 7.5E-04 | 5.8E-03 |
| FQA47_011017 | 1 | 1E-08 | 3.8E-07 |
| FQA47_019857 | 1 | 0.0E+00 | 0.0E+00 |
| FQA47_016260 | 1 | 4.7E-06 | 7.3E-05 |
| FQA47_003901 | 1 | 0.0E+00 | 0.0E+00 |
| FQA47_021114 | 1 | 1.8E-06 | 3.2E-05 |
| FQA47_011828 | 1 | 3.4E-13 | 4.2E-11 |
| FQA47_010391 | 1 | 1.0E-10 | 6.4E-09 |
| FQA47_013288 | 1 | 8.2E-09 | 2.9E-07 |
| FQA47_023680 | 2 | 1.9E-03 | 1.2E-02 |
| FQA47_004746 | 1 | 8.9E-15 | 2E-12 |
| FQA47_003573 | 1 | 8.8E-10 | 3.8E-08 |
| FQA47_004469 | 2 | 3.4E-06 | 5.5E-05 |
| FQA47_020012 | 1 | 4.0E-03 | 2.3E-02 |
| FQA47_011108 | 1 | 7.0E-06 | 1E-04 |
| FQA47_024331 | 1 | 4.4E-03 | 2.5E-02 |
| FQA47_016106 | 2 | 3.3E-08 | 9.7E-07 |
| FQA47_022487 | 3 | 8.4E-03 | 4E-02 |
| FQA47_009673 | 1 | 2.4E-08 | 7.3E-07 |
| FQA47_018707 | 1 | 1.3E-15 | 5.2E-13 |
| FQA47_013787 | 2 | 1E-06 | 2E-05 |
| FQA47_012699 | 2 | 5.5E-05 | 6.3E-04 |
| FQA47_015574 | 1 | 2.3E-05 | 3.0E-04 |
| FQA47_006037 | 1 | 3.2E-05 | 3.9E-04 |
| FQA47_012718 | 1 | 9.9E-05 | 1.0E-03 |
| FQA47_000288 | 1 | 2.3E-03 | 1.5E-02 |
| FQA47_020262 | 1 | 3E-04 | 2.8E-03 |
| FQA47_006681 | 1 | 3.7E-04 | 3.3E-03 |
| FQA47_025283 | 1 | 2.7E-06 | 4.6E-05 |
| FQA47_023536 | 1 | 3.9E-03 | 2.3E-02 |
| FQA47_013893 | 2 | 9.9E-09 | 3.4E-07 |
| FQA47_024673 | 1 | 1E-03 | 8.2E-03 |
| FQA47_008373 | 3 | 7.4E-04 | 5.7E-03 |
| FQA47_000204 | 3 | 2.2E-07 | 5.0E-06 |
| FQA47_002598 | 1 | 5.7E-05 | 6.5E-04 |
| FQA47_013690 | 1 | 9.3E-03 | 4.5E-02 |
| FQA47_006901 | 1 | 8.8E-04 | 6.5E-03 |
| FQA47_010984 | 2 | 7.6E-09 | 2.8E-07 |
| FQA47_023661 | 1 | 4.9E-03 | 2.7E-02 |
| FQA47_025335 | 1 | 5.5E-03 | 3.0E-02 |
| FQA47_008575 | 1 | 2.8E-03 | 1.7E-02 |
| FQA47_003978 | 1 | 4.5E-04 | 3.8E-03 |
| FQA47_007218 | 1 | 5.4E-14 | 9.4E-12 |
| FQA47_004709 | 1 | 9.7E-10 | 4.2E-08 |
| FQA47_000362 | 1 | 4.3E-12 | 3.8E-10 |
| FQA47_018055 | 1 | 6.5E-03 | 3.4E-02 |
| FQA47_005248 | 2 | 2.6E-05 | 3.4E-04 |
| FQA47_009074 | 1 | 2.3E-04 | 2E-03 |
| FQA47_012321 | 1 | 7E-05 | 7.8E-04 |
| FQA47_021989 | 4 | 1.3E-07 | 3.3E-06 |
| FQA47_025362 | 1 | 9.0E-06 | 1.3E-04 |
| FQA47_019365 | 2 | 6E-11 | 4.0E-09 |
| FQA47_007241 | 1 | 1.8E-03 | 1.2E-02 |
| FQA47_014821 | 2 | 4.5E-06 | 7.0E-05 |
| FQA47_004870 | 2 | 1.5E-04 | 1.5E-03 |
| FQA47_023872 | 1 | 4.5E-06 | 7.0E-05 |
| FQA47_001310 | 1 | 3.0E-10 | 1.5E-08 |
| FQA47_012260 | 1 | 2.9E-08 | 8.8E-07 |
| FQA47_016242 | 1 | 2.2E-16 | 1.2E-13 |
| FQA47_003430 | 2 | 8.5E-14 | 1.4E-11 |
| FQA47_004084 | 1 | 8.2E-09 | 2.9E-07 |
| FQA47_003435 | 1 | 7.2E-08 | 1.9E-06 |
| FQA47_015981 | 1 | 1E-05 | 1.6E-04 |
| FQA47_024573 | 1 | 1.0E-04 | 1E-03 |
| FQA47_011634 | 1 | 2.2E-04 | 2E-03 |
| FQA47_007924 | 1 | 3.4E-06 | 5.5E-05 |
| FQA47_014513 | 1 | 4.6E-03 | 2.6E-02 |
| FQA47_017789 | 1 | 1.4E-06 | 2.5E-05 |
| FQA47_012707 | 1 | 4.9E-10 | 2.4E-08 |
| FQA47_002792 | 1 | 1.7E-12 | 1.7E-10 |
| FQA47_011730 | 2 | 3E-05 | 3.8E-04 |
| FQA47_008076 | 1 | 6.3E-03 | 3.3E-02 |
| FQA47_020589 | 1 | 6.9E-03 | 3.6E-02 |
| FQA47_003824 | 1 | 1.7E-11 | 1.3E-09 |
| FQA47_001314 | 1 | 1E-08 | 3.7E-07 |
| FQA47_008808 | 1 | 1E-09 | 4.5E-08 |
| FQA47_010444 | 2 | 2.2E-16 | 1.2E-13 |
| FQA47_021713 | 2 | 3.7E-05 | 4.4E-04 |
| FQA47_009876 | 2 | 7E-07 | 1.4E-05 |
| FQA47_001495 | 4 | 1E-04 | 1.2E-03 |
| FQA47_012353 | 1 | 6.3E-07 | 1.3E-05 |
| FQA47_017984 | 1 | 8.3E-05 | 9.0E-04 |
| FQA47_014983 | 2 | 6.4E-04 | 5E-03 |

**Table S15. Gene Ontology (GO) enrichment of positively selected genes (PSGs) in the *O. melastigma.***

| **GO ID** | **Term** | **Class** | **# in PSGs** | **# in the genome** | ***P*-value** |
| --- | --- | --- | --- | --- | --- |
| GO:0003723 | RNA binding | MF | 13 | 444 | 0.001196 |
| GO:0016592 | mediator complex | CC | 3 | 25 | 0.002429 |
| GO:0015197 | peptide transporter activity | MF | 5 | 86 | 0.002457 |
| GO:0015833 | peptide transport | BP | 5 | 86 | 0.002457 |
| GO:0042886 | amide transport | BP | 5 | 89 | 0.002853 |
| GO:0019902 | phosphatase binding | MF | 2 | 9 | 0.004009 |
| GO:0000989 | transcription factor binding transcription factor activity | MF | 4 | 61 | 0.004342 |
| GO:0003712 | transcription cofactor activity | MF | 4 | 61 | 0.004342 |
| GO:0006412 | translation | BP | 11 | 429 | 0.00746 |
| GO:0004990 | oxytocin receptor activity | MF | 3 | 39 | 0.008634 |
| GO:0005198 | structural molecule activity | MF | 26 | 1434 | 0.008833 |
| GO:0009982 | pseudouridine synthase activity | MF | 2 | 14 | 0.009779 |
| GO:0071705 | nitrogen compound transport | BP | 8 | 273 | 0.010155 |
| GO:0045665 | negative regulation of neuron differentiation | BP | 1 | 1 | 0.010842 |
| GO:0050768 | negative regulation of neurogenesis | BP | 1 | 1 | 0.010842 |
| GO:0016560 | protein import into peroxisome matrix, docking | BP | 1 | 1 | 0.010842 |
| GO:0000176 | nuclear exosome (RNase complex) | CC | 1 | 1 | 0.010842 |
| GO:0004609 | phosphatidylserine decarboxylase activity | MF | 1 | 1 | 0.010842 |
| GO:0008452 | RNA ligase activity | MF | 1 | 1 | 0.010842 |
| GO:0010721 | negative regulation of cell development | BP | 1 | 1 | 0.010842 |
| GO:0022615 | protein to membrane docking | BP | 1 | 1 | 0.010842 |
| GO:0007342 | fusion of sperm to egg plasma membrane | BP | 2 | 15 | 0.011203 |
| GO:0007338 | single fertilization | BP | 2 | 15 | 0.011203 |
| GO:0009566 | fertilization | BP | 2 | 15 | 0.011203 |
| GO:0045026 | plasma membrane fusion | BP | 2 | 15 | 0.011203 |
| GO:0000988 | protein binding transcription factor activity | MF | 4 | 87 | 0.014898 |
| GO:0001522 | pseudouridine synthesis | BP | 2 | 18 | 0.01598 |
| GO:0022412 | cellular process involved in reproduction in multicellular organism | BP | 2 | 18 | 0.01598 |
| GO:0008484 | sulfuric ester hydrolase activity | MF | 2 | 19 | 0.017734 |
| GO:0005083 | small GTPase regulator activity | MF | 5 | 141 | 0.018945 |
| GO:0006357 | regulation of transcription from RNA polymerase II promoter | BP | 3 | 53 | 0.019817 |
| GO:0004827 | proline-tRNA ligase activity | MF | 1 | 2 | 0.021568 |
| GO:0006433 | prolyl-tRNA aminoacylation | BP | 1 | 2 | 0.021568 |
| GO:0016768 | spermine synthase activity | MF | 1 | 2 | 0.021568 |
| GO:0019543 | propionate catabolic process | BP | 1 | 2 | 0.021568 |
| GO:0047547 | 2-methylcitrate dehydratase activity | MF | 1 | 2 | 0.021568 |
| GO:0034773 | histone H4-K20 trimethylation | BP | 1 | 2 | 0.021568 |
| GO:0042799 | histone methyltransferase activity (H4-K20 specific) | MF | 1 | 2 | 0.021568 |
| GO:0006564 | L-serine biosynthetic process | BP | 1 | 2 | 0.021568 |
| GO:0030328 | prenylcysteine catabolic process | BP | 1 | 2 | 0.021568 |
| GO:0034464 | BBSome | CC | 1 | 2 | 0.021568 |
| GO:0019541 | propionate metabolic process | BP | 1 | 2 | 0.021568 |
| GO:0019626 | short-chain fatty acid catabolic process | BP | 1 | 2 | 0.021568 |
| GO:0046459 | short-chain fatty acid metabolic process | BP | 1 | 2 | 0.021568 |
| GO:0018023 | peptidyl-lysine trimethylation | BP | 1 | 2 | 0.021568 |
| GO:0034770 | histone H4-K20 methylation | BP | 1 | 2 | 0.021568 |
| GO:0000098 | sulfur amino acid catabolic process | BP | 1 | 2 | 0.021568 |
| GO:0030329 | prenylcysteine metabolic process | BP | 1 | 2 | 0.021568 |
| GO:0042219 | cellular modified amino acid catabolic process | BP | 1 | 2 | 0.021568 |
| GO:0044273 | sulfur compound catabolic process | BP | 1 | 2 | 0.021568 |
| GO:0006935 | chemotaxis | BP | 9 | 378 | 0.022742 |
| GO:0042330 | taxis | BP | 9 | 378 | 0.022742 |
| GO:0044801 | single-organism membrane fusion | BP | 2 | 22 | 0.023452 |
| GO:0001104 | RNA polymerase II transcription cofactor activity | MF | 2 | 24 | 0.027628 |
| GO:0005833 | hemoglobin complex | CC | 2 | 24 | 0.027628 |
| GO:0001076 | RNA polymerase II transcription factor binding transcription factor activity | MF | 2 | 24 | 0.027628 |
| GO:0050801 | ion homeostasis | BP | 8 | 329 | 0.027677 |
| GO:0055080 | cation homeostasis | BP | 8 | 329 | 0.027677 |
| GO:0003735 | structural constituent of ribosome | MF | 7 | 269 | 0.027902 |
| GO:0004896 | cytokine receptor activity | MF | 7 | 270 | 0.028391 |
| GO:0004994 | somatostatin receptor activity | MF | 4 | 107 | 0.029261 |
| GO:0003713 | transcription coactivator activity | MF | 2 | 25 | 0.029819 |
| GO:0018193 | peptidyl-amino acid modification | BP | 2 | 25 | 0.029819 |
| GO:0048878 | chemical homeostasis | BP | 8 | 337 | 0.031271 |
| GO:0006885 | regulation of pH | BP | 6 | 218 | 0.032002 |
| GO:0015385 | sodium:proton antiporter activity | MF | 6 | 218 | 0.032002 |
| GO:0055067 | monovalent inorganic cation homeostasis | BP | 6 | 218 | 0.032002 |
| GO:0005451 | monovalent cation:proton antiporter activity | MF | 6 | 218 | 0.032002 |
| GO:0006597 | spermine biosynthetic process | BP | 1 | 3 | 0.032177 |
| GO:2001070 | starch binding | MF | 1 | 3 | 0.032177 |
| GO:0017176 | phosphatidylinositol N-acetylglucosaminyltransferase activity | MF | 1 | 3 | 0.032177 |
| GO:0016197 | endosomal transport | BP | 1 | 3 | 0.032177 |
| GO:0018836 | alkylmercury lyase activity | MF | 1 | 3 | 0.032177 |
| GO:0043141 | ATP-dependent 5'-3' DNA helicase activity | MF | 1 | 3 | 0.032177 |
| GO:0046413 | organomercury catabolic process | BP | 1 | 3 | 0.032177 |
| GO:0006353 | DNA-templated transcription, termination | BP | 1 | 3 | 0.032177 |
| GO:0008215 | spermine metabolic process | BP | 1 | 3 | 0.032177 |
| GO:0045596 | negative regulation of cell differentiation | BP | 1 | 3 | 0.032177 |
| GO:0018022 | peptidyl-lysine methylation | BP | 1 | 3 | 0.032177 |
| GO:0018941 | organomercury metabolic process | BP | 1 | 3 | 0.032177 |
| GO:0018942 | organometal metabolic process | BP | 1 | 3 | 0.032177 |
| GO:0043233 | organelle lumen | CC | 6 | 221 | 0.03387 |
| GO:0070013 | intracellular organelle lumen | CC | 6 | 221 | 0.03387 |
| GO:0048037 | cofactor binding | MF | 6 | 222 | 0.034509 |
| GO:0031974 | membrane-enclosed lumen | CC | 6 | 222 | 0.034509 |
| GO:0050662 | coenzyme binding | MF | 5 | 167 | 0.035749 |
| GO:0004950 | chemokine receptor activity | MF | 6 | 224 | 0.035808 |
| GO:0001637 | G-protein coupled chemoattractant receptor activity | MF | 6 | 224 | 0.035808 |
| GO:0038046 | enkephalin receptor activity | MF | 2 | 28 | 0.036786 |
| GO:0016866 | intramolecular transferase activity | MF | 2 | 28 | 0.036786 |
| GO:0005840 | ribosome | CC | 7 | 286 | 0.037012 |
| GO:0015491 | cation:cation antiporter activity | MF | 6 | 226 | 0.037139 |
| GO:0015299 | solute:proton antiporter activity | MF | 6 | 230 | 0.039895 |
| GO:0044763 | single-organism cellular process | BP | 98 | 10580 | 0.040489 |
| GO:0003684 | damaged DNA binding | MF | 2 | 30 | 0.041742 |
| GO:0032504 | multicellular organism reproduction | BP | 2 | 30 | 0.041742 |
| GO:0044702 | single organism reproductive process | BP | 2 | 30 | 0.041742 |
| GO:0000042 | protein targeting to Golgi | BP | 1 | 4 | 0.042672 |
| GO:0015018 | galactosylgalactosylxylosylprotein 3-beta-glucuronosyltransferase activity | MF | 1 | 4 | 0.042672 |
| GO:0008762 | UDP-N-acetylmuramate dehydrogenase activity | MF | 1 | 4 | 0.042672 |
| GO:0008241 | peptidyl-dipeptidase activity | MF | 1 | 4 | 0.042672 |
| GO:0004816 | asparagine-tRNA ligase activity | MF | 1 | 4 | 0.042672 |
| GO:0006421 | asparaginyl-tRNA aminoacylation | BP | 1 | 4 | 0.042672 |
| GO:0009062 | fatty acid catabolic process | BP | 1 | 4 | 0.042672 |
| GO:0072329 | monocarboxylic acid catabolic process | BP | 1 | 4 | 0.042672 |
| GO:0000301 | retrograde transport, vesicle recycling within Golgi | BP | 1 | 4 | 0.042672 |
| GO:0034067 | protein localization to Golgi apparatus | BP | 1 | 4 | 0.042672 |
| GO:0072600 | establishment of protein localization to Golgi | BP | 1 | 4 | 0.042672 |
| GO:0015020 | glucuronosyltransferase activity | MF | 1 | 4 | 0.042672 |
| GO:0043139 | 5'-3' DNA helicase activity | MF | 1 | 4 | 0.042672 |
| GO:0000178 | exosome (RNase complex) | CC | 1 | 4 | 0.042672 |
| GO:0015298 | solute:cation antiporter activity | MF | 6 | 238 | 0.045787 |
| GO:0031981 | nuclear lumen | CC | 5 | 179 | 0.045857 |
| GO:0004003 | ATP-dependent DNA helicase activity | MF | 2 | 32 | 0.046932 |
| GO:0006366 | transcription from RNA polymerase II promoter | BP | 3 | 75 | 0.048109 |
| GO:0004527 | exonuclease activity | MF | 2 | 33 | 0.049612 |
| GO:0006605 | protein targeting | BP | 3 | 76 | 0.049699 |

**Table S16. KEGG pathway descriptions of those positively selected genes in *O. melastigma*, which showed significant *P-*value (0.05).**

| ***O. melastigma* gene locus tag** | **Sites** | ***P*-value** | **Adj. *P*-value** | **Gene names** | **KEGG description** |  |  |  |  |
| --- | --- | --- | --- | --- | --- | --- | --- | --- | --- |
| FQA47_000976 | 1 | 1.3E-05 | 1.8E-04 | *srr* | threo-3-hydroxyaspartate ammonia-lyase-like; K01754 threonine dehydratase [EC:4.39] |  |  |  |  |
| FQA47_001654 | 1 | 2E-09 | 8.3E-08 | *Psat1* | psat1; phosphoserine aminotransferase 1; K00831 phosphoserine aminotransferase [EC:2.6.52] |  |  |  |  |
| FQA47_021114 | 1 | 1.8E-06 | 3.2E-05 | *PIPOX* | pipox; pipecolic acid oxidase; K00306 sarcosine oxidase / L-pipecolate oxidase [EC:1.5.3 1.5.3.7] |  |  |  |  |
| FQA47_021713 | 2 | 3.7E-05 | 4.4E-04 | *GCAT* | gcat; glycine C-acetyltransferase; K00639 glycine C-acetyltransferase [EC:2.3.29] |  |  |  |  |
| FQA47_013713 | 1 | 9E-05 | 9.8E-04 | *GRHPR* | glyoxylate reductase/hydroxypyruvate reductase-like; K00049 glyoxylate/hydroxypyruvate reductase [EC:1.79 1.81] |  |  |  |  |
| FQA47_003824 | 1 | 1.7E-11 | 1.3E-09 | *Sf3a3* | sf3a3; splicing factor 3a, subunit 3, 60kDa; K12827 splicing factor 3A subunit 3 |  |  |  |  |
| FQA47_012793 | 1 | 8.9E-08 | 2.3E-06 | *alyref-a* | THO complex subunit 4-like; K12881 THO complex subunit 4 |  |  |  |  |
| FQA47_008100 | 2 | 1.5E-08 | 4.9E-07 | *Snrpa1* | snrpa1; small nuclear ribonucleoprotein polypeptide A'; K11092 U2 small nuclear ribonucleoprotein A' |  |  |  |  |
| FQA47_008513 | 1 | 1E-05 | 1.5E-04 | *snrnp27* | snrnp27; small nuclear ribonucleoprotein 27kDa (U4/U6.U5); K12846 U4/U6.U5 tri-snRNP-associated protein 3 |  |  |  |  |
| FQA47_011754 | 1 | 1.2E-07 | 3.0E-06 | *EFHC1* | hypothetical protein; K12847 U4/U6.U5 tri-snRNP-associated protein 2 |  |  |  |  |
| FQA47_013043 | 1 | 1.0E-02 | 4.8E-02 | *Thoc3* | thoc3; THO complex 3; K12880 THO complex subunit 3 |  |  |  |  |
| FQA47_024405 | 1 | 2.0E-06 | 3.4E-05 | *HNRNPM* | hnrnpm; heterogeneous nuclear ribonucleoprotein M; K12887 heterogeneous nuclear ribonucleoprotein M |  |  |  |  |
| FQA47_022520 | 1 | 7.4E-04 | 5.8E-03 | *PIGH* | pigh; phosphatidylinositol glycan anchor biosynthesis, class H; K03858 phosphatidylinositol glycan, class H |  |  |  |  |
| FQA47_023891 | 1 | 6E-15 | 1.5E-12 | *Pigl* | pigl; phosphatidylinositol glycan anchor biosynthesis, class L; K03434 N-acetylglucosaminylphosphatidylinositol deacetylase [EC:3.5.89] |  |  |  |  |
| FQA47_006534 | 1 | 8.6E-04 | 6.4E-03 | *pigb* | pigb; phosphatidylinositol glycan anchor biosynthesis, class B; K05286 phosphatidylinositol glycan, class B [EC:2.4.-] |  |  |  |  |
| FQA47_005935 | 1 | 3.3E-16 | 1.7E-13 | *ddb2* | ddb2; damage-specific DNA binding protein 2, 48kDa; K10140 DNA damage-binding protein 2 |  |  |  |  |
| FQA47_012111 | 1 | 6.8E-03 | 3.5E-02 | *fahd2* | hypothetical protein; K03142 transcription initiation factor TFIIH subunit 2 |  |  |  |  |
| FQA47_018400 | 1 | 1.5E-07 | 3.7E-06 | *RAD23B* | rad23b; RAD23 homolog B (S. cerevisiae); K10839 UV excision repair protein RAD23 |  |  |  |  |
| FQA47_025458 | 1 | 6.6E-04 | 5.2E-03 | *Rbx1* | rbx1; ring-box 1, E3 ubiquitin protein ligase; K03868 RING-box protein 1 |  |  |  |  |
| FQA47_000178 | 1 | 4E-04 | 3.5E-03 | *VAMP8* | vesicle-associated membrane protein 8-like; K08512 vesicle-associated membrane protein 8 |  |  |  |  |
| FQA47_006037 | 1 | 3.2E-05 | 3.9E-04 | *STX5* | stx5; syntaxin 5; K08490 syntaxin 5 | | | |  |
| FQA47_008373 | 3 | 7.4E-04 | 5.7E-03 | *Snap23* | snap23; synaptosomal-associated protein, 23kDa; K08508 synaptosomal-associated protein 23 |  |  |  |  |
| FQA47_015151 | 1 | 7.3E-03 | 3.7E-02 | *CD36* | cd36; CD36 molecule (thrombospondin receptor); K06259 CD36 antigen |  |  |  |  |
| FQA47_012217 | 1 | 2.2E-03 | 1.4E-02 | *HMCN1* | B-cell receptor CD22-like; K06467 CD22 antigen |  |  |  |  |
| FQA47_010675 | 1 | 3.2E-03 | 1.9E-02 | *-* | CD7; CD7 molecule; K06457 CD7 antigen |  |  |  |  |
| FQA47_018866 | 1 | 2.9E-03 | 1.8E-02 | *-* | uncharacterized LOC101157618; K06474 CD34 antigen |  |  |  |  |
| FQA47_002792 | 1 | 1.7E-12 | 1.7E-10 | *Tfr2* | tfrc; transferrin receptor; K06503 transferrin receptor |  |  |  |  |
| FQA47_005935 | 1 | 3.3E-16 | 1.7E-13 | *ddb2* | ddb2; damage-specific DNA binding protein 2, 48kDa; K10140 DNA damage-binding protein 2 |  |  |  |  |
| FQA47_013680 | 1 | 1.8E-05 | 2.4E-04 | *-* | gtse1; G-2 and S-phase expressed 1; K10129 G-2 and S-phase expressed protein 1 |  |  |  |  |
| FQA47_022987 | 1 | 2.4E-04 | 2.2E-03 | *RFWD2* | rfwd2; ring finger and WD repeat domain 2, E3 ubiquitin protein ligase; K10143 E3 ubiquitin-protein ligase RFWD2 [EC:2.3.2.27] |  |  |  |  |
| FQA47_013893 | 2 | 9.9E-09 | 3.4E-07 | *Cd82* | cd82; CD82 molecule; K06509 CD82 antigen |  |  |  |  |
| FQA47_018055 | 1 | 6.5E-03 | 3.4E-02 | *ccnb2* | ccnb2; cyclin B2; K05868 cyclin B | | | |  |
| FQA47_000976 | 1 | 1.3E-05 | 1.8E-04 | *srr* | threo-3-hydroxyaspartate ammonia-lyase-like; K01754 threonine dehydratase [EC:4.39] |  |  |  |  |
| FQA47_001654 | 1 | 2E-09 | 8.3E-08 | *Psat1* | psat1; phosphoserine aminotransferase 1; K00831 phosphoserine aminotransferase [EC:2.6.52] |  |  |  |  |
| FQA47_001618 | 1 | 8.9E-16 | 3.7E-13 | *Rpe* | rpe; ribulose-5-phosphate-3-epimerase; K01783 ribulose-phosphate 3-epimerase [EC:5.3] |  |  |  |  |
| FQA47_012795 | 2 | 1.2E-03 | 8.5E-03 | *myadm* | PYCR1; pyrroline-5-carboxylate reductase 1; K00286 pyrroline-5-carboxylate reductase [EC:1.5.2] |  |  |  |  |
| FQA47_008808 | 1 | 1E-09 | 4.5E-08 | *chst11* | chst11; carbohydrate (chondroitin 4) sulfotransferase 11; K01017 chondroitin 4-sulfotransferase 11 [EC:2.8.2.5] |  |  |  |  |
| FQA47_025472 | 2 | 3.2E-09 | 1.2E-07 | *TOP3A* | top3a; topoisomerase (DNA) III alpha; K03165 DNA topoisomerase III [EC:5.99.2] |  |  |  |  |
| FQA47_014093 | 1 | 1.9E-07 | 4.3E-06 | *mre11* | mre11a; MRE11 meiotic recombination 11 homolog A (S. cerevisiae); K10865 double-strand break repair protein MRE11 |  |  |  |  |
| FQA47_002466 | 1 | 3.4E-03 | 2.0E-02 | *Xrcc3* | xrcc3; X-ray repair complementing defective repair in Chinese hamster cells 3; K10880 DNA-repair protein XRCC3 |  |  |  |  |
| FQA47_001266 | 1 | 8.0E-03 | 4.0E-02 | *DGKE* | dgke; diacylglycerol kinase, epsilon 64kDa; K00901 diacylglycerol kinase (ATP) [EC:2.707] |  |  |  |  |
| FQA47_003732 | 2 | 2.3E-03 | 1.5E-02 | *AGPAT1* | agpat2; 1-acylglycerol-3-phosphate O-acyltransferase 2; K13509 lysophosphatidate acyltransferase [EC:2.3.51] |  |  |  |  |
| FQA47_011108 | 1 | 7.0E-06 | 1E-04 | *PISD* | pisd; phosphatidylserine decarboxylase; K01613 phosphatidylserine decarboxylase [EC:4.65] |  |  |  |  |
| FQA47_024331 | 1 | 4.4E-03 | 2.5E-02 | *GPCPD1* | gpcpd1; glycerophosphocholine phosphodiesterase GDE1 homolog (S. cerevisiae); K18695 glycerophosphocholine phosphodiesterase GPCPD1 [EC:3.4.2] |  |  |  |  |
| FQA47_006681 | 1 | 3.7E-04 | 3.3E-03 | *VPS39* | PLA2G4F; uncharacterized LOC100543562; K16342 cytosolic phospholipase A2 [EC:3.4] |  |  |  |  |
| FQA47_020210 | 1 | 9.8E-06 | 1.4E-04 | *MRPL3* | mrpl3; mitochondrial ribosomal protein L3; K02906 large subunit ribosomal protein L3 |  |  |  |  |
| FQA47_017682 | 1 | 3.9E-04 | 3.4E-03 | *RPL29* | rpl29; ribosomal protein L29; K02905 large subunit ribosomal protein L29e |  |  |  |  |
| FQA47_012325 | 1 | 7.5E-04 | 5.8E-03 | *rpl36a* | RPL36A; ribosomal protein L36a; K02929 large subunit ribosomal protein L44e |  |  |  |  |
| FQA47_011730 | 2 | 3E-05 | 3.8E-04 | *MRPL19* | mrpl19; mitochondrial ribosomal protein L19; K02884 large subunit ribosomal protein L19 |  |  |  |  |
| FQA47_020589 | 1 | 6.9E-03 | 3.6E-02 | *rpl9* | rpl9; ribosomal protein L9; K02940 large subunit ribosomal protein L9e |  |  |  |  |

**Table S17. Species included in the comparative genomics in this study.**

| **Species** | **Genome resource** | **Annotation resource** |
| --- | --- | --- |
| *Oryzias latipes* | ftp://ftp.ensembl.org/pub/release-92/fasta/oryzias_latipes/dna/Oryzias_latipes.MEDAKA1.dna.toplevel.fa.gz | ftp://ftp.ensembl.org/pub/release-92/gff3/oryzias_latipes/Oryzias_latipes.MEDAKA1.92.gff3.gz |
| *Xiphophorus maculatus* | ftp://ftp.ensembl.org/pub/release-92/fasta/xiphophorus_maculatus/dna/Xiphophorus_maculatus.Xipmac4.4.2.dna.toplevel.fa.gz | ftp://ftp.ensembl.org/pub/release-92/gff3/xiphophorus_maculatus/Xiphophorus_maculatus.Xipmac4.4.2.92.gff3.gz |
| *Nothobranchius furzeri* | https://ftp.ncbi.nlm.nih.gov/genomes/all/GCF/001/465/895/GCF_001465895.1_Nfu_20140520/GCF_001465895.1_Nfu_20140520_genomic.fna.gz | https://ftp.ncbi.nlm.nih.gov/genomes/all/GCF/001/465/895/GCF_001465895.1_Nfu_20140520/GCF_001465895.1_Nfu_20140520_genomic.gff.gz |
| *Oreochromis niloticus* | ftp://ftp.ensembl.org/pub/release-92/fasta/oreochromis_niloticus/dna/Oreochromis_niloticus.Orenil1.0.dna.toplevel.fa.gz | ftp://ftp.ensembl.org/pub/release-92/gff3/oreochromis_niloticus/Oreochromis_niloticus.Orenil1.0.92.gff3.gz |
| *Takifugu rubripes* | ftp://ftp.ensembl.org/pub/release-92/fasta/takifugu_rubripes/dna/Takifugu_rubripes.FUGU4.dna.toplevel.fa.gz | ftp://ftp.ensembl.org/pub/release-92/gff3/takifugu_rubripes/Takifugu_rubripes.FUGU4.92.gff3.gz |
| *Tetraodon nigroviridis* | ftp://ftp.ensembl.org/pub/release-92/fasta/tetraodon_nigroviridis/dna/Tetraodon_nigroviridis.TETRAODON8.dna.toplevel.fa.gz | ftp://ftp.ensembl.org/pub/release-92/gff3/tetraodon_nigroviridis/Tetraodon_nigroviridis.TETRAODON8.92.gff3.gz |
| *Gasterosteus aculeatus* | ftp://ftp.ensembl.org/pub/release-92/fasta/gasterosteus_aculeatus/dna/Gasterosteus_aculeatus.BROADS1.dna.toplevel.fa.gz | ftp://ftp.ensembl.org/pub/release-92/gff3/gasterosteus_aculeatus/Gasterosteus_aculeatus.BROADS1.dna.toplevel.fa.gz |
| *Gadus morhua* | ftp://ftp.ensembl.org/pub/release-92/fasta/gadus_morhua/dna/Gadus_morhua.gadMor1.dna.toplevel.fa.gz | ftp://ftp.ensembl.org/pub/release-92/gff3/gadus_morhua/Gadus_morhua.gadMor1.92.gff3.gz |
| *Salmo salar* | https://ftp.ncbi.nlm.nih.gov/genomes/all/GCF/000/233/375/GCF_000233375.1_ICSASG_v2/GCF_000233375.1_ICSASG_v2_genomic.fna.gz | https://ftp.ncbi.nlm.nih.gov/genomes/all/GCF/000/233/375/GCF_000233375.1_ICSASG_v2/GCF_000233375.1_ICSASG_v2_genomic.gff.gz |
| *Ctenopharyngodon idellus* | http://www.ncgr.ac.cn/grasscarp/files/C_idella_female_scaffolds.fasta.v1.gz | http://www.ncgr.ac.cn/grasscarp/files/C_idella_female_genemodels.v1.gmap.gff3.gz |
| *Cyprinus carpio* | https://ftp.ncbi.nlm.nih.gov/genomes/all/GCF/000/951/615/GCF_000951615.1_common_carp_genome/GCF_000951615.1_common_carp_genome_genomic.fna.gz | https://ftp.ncbi.nlm.nih.gov/genomes/all/GCF/000/951/615/GCF_000951615.1_common_carp_genome/GCF_000951615.1_common_carp_genome_genomic.gff.gz |
| *Danio rerio* | ftp://ftp.ensembl.org/pub/release-92/fasta/danio_rerio/dna/Danio_rerio.GRCz11.dna.toplevel.fa.gz | ftp://ftp.ensembl.org/pub/release-92/gff3/danio_rerio/Danio_rerio.GRCz11.92.gff3.gz |
| *Ictalurus punctatus* | https://ftp.ncbi.nlm.nih.gov/genomes/all/GCF/001/660/625/GCF_001660625.1_IpCoco_1.2/GCF_001660625.1_IpCoco_1.2_genomic.fna.gz | https://ftp.ncbi.nlm.nih.gov/genomes/all/GCF/001/660/625/GCF_001660625.1_IpCoco_1.2/GCF_001660625.1_IpCoco_1.2_genomic.gff.gz |
| *Electrophorus electricus* | https://efishgenomics.integrativebiology.msu.edu/downloads/Genome-Ee/SOAPdenovo-genome.fa.gz | https://efishgenomics.integrativebiology.msu.edu/downloads/Genome-Ee/Ee_soap_1.4.gff3.gz |
| *Astyanax mexicanus* | ftp://ftp.ensembl.org/pub/release-92/fasta/astyanax_mexicanus/dna/Astyanax_mexicanus.AstMex102.dna.toplevel.fa.gz | ftp://ftp.ensembl.org/pub/release-92/gff3/astyanax_mexicanus/Astyanax_mexicanus.AstMex102.92.gff3.gz |
| *Lepisosteus oculatus* | ftp://ftp.ensembl.org/pub/release-92/fasta/lepisosteus_oculatus/dna/Lepisosteus_oculatus.LepOcu1.dna.toplevel.fa.gz | ftp://ftp.ensembl.org/pub/release-92/gff3/lepisosteus_oculatus/Lepisosteus_oculatus.LepOcu1.92.gff3.gz |
| *Homo sapiens* | ftp://ftp.ensembl.org/pub/release-92/fasta/homo_sapiens/dna/Homo_sapiens.GRCh38.dna.toplevel.fa.gz | ftp://ftp.ensembl.org/pub/release-92/gff3/homo_sapiens/Homo_sapiens.GRCh38.92.gff3.gz |
